# Model-Based Characterization of Inflammatory Gene Expression Patterns of Activated Macrophages

**DOI:** 10.1371/journal.pcbi.1005018

**Published:** 2016-07-27

**Authors:** Julia Rex, Ute Albrecht, Christian Ehlting, Maria Thomas, Ulrich M. Zanger, Oliver Sawodny, Dieter Häussinger, Michael Ederer, Ronny Feuer, Johannes G. Bode

**Affiliations:** 1 Institute for System Dynamics, University of Stuttgart, Stuttgart, Germany; 2 Clinic of Gastroenterology, Hepatology and Infectious Diseases, Heinrich-Heine-University, Düsseldorf, Germany; 3 Dr. Margarete Fischer-Bosch Institute of Clinical Pharmacology, Stuttgart, and University of Tübingen, Tübingen, Germany; University of California San Diego, UNITED STATES

## Abstract

Macrophages are cells with remarkable plasticity. They integrate signals from their microenvironment leading to context-dependent polarization into classically (M1) or alternatively (M2) activated macrophages, representing two extremes of a broad spectrum of divergent phenotypes. Thereby, macrophages deliver protective and pro-regenerative signals towards injured tissue but, depending on the eliciting damage, may also be responsible for the generation and aggravation of tissue injury. Although incompletely understood, there is emerging evidence that macrophage polarization is critical for these antagonistic roles. To identify activation-specific expression patterns of chemokines and cytokines that may confer these distinct effects a systems biology approach was applied. A comprehensive literature-based Boolean model was developed to describe the M1 (LPS-activated) and M2 (IL-4/13-activated) polarization types. The model was validated using high-throughput transcript expression data from murine bone marrow derived macrophages. By dynamic modeling of gene expression, the chronology of pathway activation and autocrine signaling was estimated. Our results provide a deepened understanding of the physiological balance leading to M1/M2 activation, indicating the relevance of co-regulatory signals at the level of Akt1 or Akt2 that may be important for directing macrophage polarization.

## Author Summary

Macrophages are essential cells of the immune system and indispensable for a defense against bacterial infection. They reside as resting, immune modulatory cells in several tissues of the human body where they continuously sense inputs from their local environment. They react to stimuli such as toxins, injury or bacterial products in a process termed macrophage activation or polarization. For example, the bacterial component lipopolysaccharide induces so-called classical activation of macrophages into the M1 phenotype that secretes a number of inflammatory cytokines and chemokines leading to killing of bacteria and resolution of inflammation. Another prominent phenotype of macrophages is the M2 polarization state that is associated with wound healing and tissue regeneration. Unbalanced activation of macrophages is implicated in a number of diseases. An improved knowledge and extensive characterization of these macrophages as well as the factors determining their phenotypes will improve the understanding of the role of macrophages in disease progression.

## Introduction

A large part of macrophage populations originate from monocytes released from the bone marrow that upon injury or inflammation migrate into several tissues of the body such as lung, liver, spleen, lymph node, bone or the central nervous system [1, 2]. As it harbors the body’s largest pool of sessile tissue macrophages the liver owns a distinguished role within the macrophage system. Lineage tracing experiments [3, 4] suggest that under homeostatic conditions macrophages recruited from circulating monocytes are of minor importance for maintenance of the population of tissue resident liver macrophages (also termed as Kupffer cells). These cells have janitorial and immune regulatory functions but play a subordinate role for induction and regulation of inflammatory reactions evoked by tissue injury. The latter is mainly adopted by macrophages recruited to the liver from circulating monocytes. They play a critical role for the generation and control of inflammatory reactions that either cause and aggravate tissue injury or mediate processes required for tissue repair [3], which are also the basis for the unique capability of the liver to regenerate [5].

These diverging functions of macrophages, being either responsible for induction and aggravation of tissue injury or for induction and maintenance of repair processes, are attributed to the fact that macrophages exhibit a remarkable plasticity and can embrace a large spectrum of different activations states and functions. They can be activated by a variety of external stimuli including microbial products, nucleotide derivatives, growth factors, glucocorticoids as well as cytokines and change their physiology in response to these environmental factors [6]. Macrophages are classified into classically activated macrophages (M1) or alternatively activated macrophages (M2) [7] representing the two poles of a continuous spectrum of polarization states [8]. In accordance with experimental guidelines that have been published recently, the M1 polarization of macrophages can be triggered by stimulation with lipopolysaccharide (LPS) and/or IFNγ [6]. These cells exhibit microbicidal or tumoricidal activity, and secrete high amounts of pro-inflammatory cytokines and upregulate the surface marker CD69. Contrariwise, M2 polarization of macrophages can be induced by stimulation with the two interleukins (IL) IL-4 and IL-13. Among others, these cells are characterized by enhanced expression of the mannose receptor (CD206) and by increased arginase activity [1, 9, 10].

The toll-like receptor 4 (TLR4) pathway in M1 macrophages that is induced in response to LPS stimulation is quite well characterized [11, 12]. It primarily leads to activation of the transcription factors NF-κB and interferon regulatory factor 3 (IRF3) [11] which in turn are involved in the regulation of gene expression including that of interferons [13], pro-inflammatory cytokines such as IL-1β, IL-6 and TNFα [14, 15] and chemokines [16]. Other key proteins that regulate amount and duration of transcript expression during an inflammatory response towards bacterial or viral infections are the p38 mitogen-activated protein kinase (p38^MAPK^) as well as the p38^MAPK^-activated protein kinases MK2 and MK3 [15, 17–22]. For a functional inflammatory response, LPS needs to successively trigger autocrine and paracrine signaling events that are critical for regulation of the initiation, the propagation and, finally, also the resolution of an inflammatory response. A cytokine which is critically involved in the resolution of an inflammatory response and controlled via such autocrine cycles is the anti-inflammatory cytokine IL-10. Lack of this cytokine or specific deletion of its major effector protein, the transcription factor (TF) signal transducer and activator of transcription (STAT)3 in macrophages [15, 23–25], results in an overwhelming and deleterious inflammatory response towards pathogens or pathogen stimuli such as LPS. The expression of this cytokine and subsequent activation of STAT3 requires the auto- and/or paracrine activation of the type I interferon receptor (IFNAR) via LPS-induced release of type I interferons (IFN) such as IFNβ. Activation of IFNAR has been demonstrated to be a prerequisite for LPS-inducible expression of interleukin 10 (IL-10) and subsequent activation of STAT3 [15, 17, 26]. The molecular mechanisms involved in the induction of alternatively activated M2 macrophages by IL-4 and IL-13 are less well characterized. Identification of the mannose receptor as a marker protein for alternative activation of macrophages was first proposed by Stein et al. [9]. Apart from this receptor, arginase 1 (Arg1), the chitinase-like molecules Ym1 and Ym2, as well as Fizz1 [27] have been identified as genes characterizing the M2 phenotype. M2 macrophages are generally involved in homeostasis, wound healing and tissue repair [28], but are also implicated in pathological processes like asthma and allergy [29]. The phosphatidylinositol 3-kinase (PI3K)/Akt pathway is activated both after stimulation with LPS [30–32] and IL-4/13 [33]. Knockout studies demonstrated an important role of the two isoforms Akt1 and Akt2 in macrophage polarization [34, 35].

To illustrate the relevance of macrophage polarization, the liver may be a good example as it clearly is central for the control of the systemic acute phase response. The maintenance of a critical balance of pro-survival and pro-apoptotic as well as pro- and anti-inflammatory signals in the liver is essential as dysregulation can have detrimental effects and might lead to organ damage [36]. Risk factors such as alcohol abuse, intake of drugs or toxins, obesity, and chronic HBV/HCV infection can disturb this balance and might develop into chronic hepatitis leading to liver fibrosis and, in the final stage, liver cirrhosis and organ failure [37]. All these conditions involve chronic activation of inflammation and, as a result of ongoing inflammation, impaired wound healing. In this context, the activation of inflammatory pathways in the liver and the release of cytokines from inflammatory cell populations, including macrophages, and their implications on hepatocytes are of particular importance [38, 39]. For example, the cytokines IL-1β and TNFα not only promote inflammation, but also influence cell death signaling in hepatocytes [40, 41]. Depending on the eliciting damage, most of the cytokines and pathways involved exhibit dual functions. For instance, NF-κB has been identified as a pathway, which mediates effects important for hepatocyte survival, but has been also identified as a signaling intermediate that propagates liver damage upon ischemia-reperfusion (I/R) injury [38]. Likewise, chemokines, such as Cxcl2 have controversial functions in the regulation of liver regeneration. It supports liver regeneration after partial hepatectomy, whereas it impairs recovery from I/R injury [42, 43]. Similarly, macrophage activation into the various phenotypes mediate heterogeneous effects. Overwhelming M1 activation can induce sepsis, predispose surrounding tissue for neoplastic transformation as well as promote insulin resistance and type 2 diabetes. Whereas excessive M2 activation can induce liver fibrosis during chronic infection and aggravate allergic responses [1]. A systematic understanding and description of the influences leading to activation of macrophages into the various phenotypes as well as the corresponding cytokine pattern is of particular importance for understanding the role of macrophages in health and disease.

In this work, we present a large-scale Boolean model that describes the major signaling pathways in M1 (LPS-activated) and M2 (IL-4/13-activated) primary murine bone marrow derived macrophages (BMDMs) as well as a dynamic model of the gene expression module. The initial literature-based network structure is adapted using high-throughput gene expression data from primary murine BMDMs in which, according to recent suggestions, M1 polarization was induced by treatment with LPS whereas M2 polarization was triggered by exposure towards IL-4 and IL-13. In addition, the dynamics of mRNA expression are investigated in more detail using an ordinary differential equation-based model to disentangle the chronology of direct signaling events and autocrine feedback loops. By estimating the time frame of transcription factor activity, the model structure is further refined, for example IL-10 appears to be upregulated before the transcription factor Stat3 is activated, suggesting that Stat3 may not be essentially required for LPS-inducible expression of IL-10. Investigation of secreted cytokines and autocrine feedback loops results in the last model refinement, that is the major pro-inflammatory cytokines inducing the acute phase response in hepatocytes, the interleukins IL-1β and IL-6 as well as TNFα [15, 36], exert no or minor autocrine effects on macrophages regarding the gene expression profile.

## Results

### Boolean model of classically and alternatively activated macrophages

For a first description of macrophage activation, we constructed a comprehensive large-scale Boolean model including relevant signaling pathways activated in macrophages upon induction of M1 polarization by stimulation with LPS or induction of a M2 phenotype in response to combined stimulation with IL-4 and IL-13 (S1 Fig). The model was based on extensive literature research and its first version contained 148 nodes and 176 interactions. Abbreviations of the network nodes are explained in S1 and S2 Tables. The model was implemented using the MATLAB Toolbox CellNetAnalyzer (CNA) [50]. It contains three input nodes (black), namely LPS and the interleukins IL-4 and IL-13. Output of the model is the gene expression pattern that results from the respective stimulation. A number of species (grey) have been demonstrated to induce autocrine feedback loops after secretion by macrophages. Among many others, this includes the cytokines IL-1β, IL-6, IL-10 and TNFα as well as the interferons IFNα, IFNβ, and IFNγ [15, 54–56].

The following signaling events are reproduced by the Boolean model: (1) LPS stimulation results in activation and formation of the TLR4 receptor complex containing TLR4, MD-2 and CD14 as well as LPS and the lipopolysaccharide binding protein (LBP). The activated TLR4 receptor complex can signal via the MyD88-dependent signaling pathway resulting in early activation of NF-κB and the mitogen-activated protein (MAP) kinases p38^MAPK^, JNK, and ERK, as well as the MyD88-independent, TRAM-/TRIF-dependent signaling pathway accounting for late phase NF-κB activation and activation of the transcription factor IRF3 [11, 12]. Furthermore, the activated TLR4 complex leads to activation of the PI3K/Akt pathway [32] and increased expression of the microRNA miRNA-155 enhancing NF-κB activity [34, 35]. The transcription factors NF-κB and IRF3 mediate expression of a number of genes associated with the classical M1 phenotype of macrophages. After secretion, some of these proteins induce autocrine signaling: (1.1) IL-1β binds to the IL-1R and its downstream signaling pathway shares similarity with TLR4-mediated signaling. Thus IL-1β also results in activation of NF-κB and the MAP kinases [54]. (1.2) Binding of TNFα to the TNFR1 results in assembly of the receptor complex 1 including TRADD, TRAF2 and Rip1. This activates the kinase TAK1 leading to activation of NF-κB and the MAP kinases similar to TLR4 signaling [54]. (1.3) IL-6 signals through its receptor consisting of the ligand binding domain gp80 and the signaling subunit gp130 that subsequently gets phosphorylated by the associated Janus kinase 1 (Jak1) leading to recruitment and activation of the TF Stat3 [57]. Activation of STAT3 is inhibited by suppressor of cytokine signaling (SOCS) 3 a member of a family of endogenous feedback inhibitors of STAT signaling, which can be induced by STAT family members themselves but also by other pathways, including the p38^MAPK^/MK2 pathway [58, 59]. (1.4) The type II interferon IFNγ binds to its receptor IFNGR resulting in activation of the TF Stat1 via Jak1 or Jak2 [56]. (1.5) The type I interferons signal via the IFNAR leading to activation of the Stat transcription factors Stat1 and Stat3 followed by expression of respective target genes [55]. (1.6) One of the target genes upregulated upon activation of IFNAR1 is IL-10 that also acts in an autocrine feedback loop via the IL-10R mediating late and sustained activation of Stat3 that, in contrast to the activation of STAT3 by other cytokines, is resistant to the action of SOCS3 [60], and important for the resolution phase of the inflammatory response [15].

(2) Stimulation with IL-4 and IL-13 results in activation of various receptor complexes. Both cytokines share a common receptor subunit, the IL-4Rα chain, which can either engage with the common γ chain (IL-2Rγ) upon IL-4 binding or with the IL-13Rα1 chain upon IL-13 binding [33]. IL-13 can also bind to the IL-13Rα2 whose cytoplasmic domains do not contain any Jak/Stat binding sequences [61] and thus is likely to act as a decoy receptor congruently with the phenotype of IL-13Rα2-deficient mice being consistent with enhanced IL-13 responsiveness [62]. IL-4/IL-13 signaling results 1 (Arg1) and mannose receptor type 1 (Mrc1) [27, 63]. In addition, binding of IL-4 to IL-4Rα leads to activation of the PI3K/Akt pathway [64] resulting in decreased expression of the microRNA miRNA-155 and increased expression of the M2 marker gene Arg1 [34, 35].

The PI3K pathway and the two isoforms Akt1 and Akt2 differentially influence macrophage polarization [34, 35]. Arranz et al. [34] demonstrated that Akt1 KO gives rise to an M1 phenotype whereas Akt2 KO induces M2 polarization accompanied by high levels of Arg1. Furthermore, they showed that downregulation of the microRNA miRNA-155 in Akt2 KO macrophages leads to elevated levels of the CCAAT/enhancer binding protein beta (C/EBPβ) and enhanced binding to the Arg1 promotor while Stat6 phosphorylation remains unaffected. In contrast, Akt1 KO leads to elevated levels of miRNA-155 which contributes to NF-κB activity and M1 polarization [35]. However, both isoforms, Akt1 and Akt2, are concurrently activated in the WT after LPS as well as IL-4/13 stimulation and differentially influence miRNA-155 expression. For correct polarization of macrophages, i. e. induction of the M1 phenotype after LPS stimulation and induction of the M2 phenotype following IL-4/13 stimulation, the introduction of an additional signal into the model was necessary. This coregulating signal is induced by the activated TLR4 receptor complex and acts on the level of Akt activation or downstream signaling. Otherwise, the positive influence of Akt2 on miRNA-155 expression overcomes the inhibitory influence mediated by Akt1 leading to induction of the M1 phenotype even after IL-4/13 stimulation (S2 Fig). Indeed, we observed increased expression of miRNA-155 after 6 h treatment of BMDMs with LPS and decreased expression after 6 h IL-4/IL-13 stimulation (S3 Fig).

### Extension of the logical formalism: timescale constants

In order to reproduce the sequential release of various factors by macrophages and following induction of autocrine signaling loops, timescale constants are introduced to the Boolean model [49]. A so called timescale constant τ can be assigned to all logical interactions as a parameter that specifies when a distinct signaling event takes place. A lower timescale constant indicates that the reaction becomes active earlier than an interaction with a higher timescale constant.

The model introduced here contains eight timescales τ = {0, 1, 2, 5, 7, 10, 12, 15} that are used to separate distinct signaling events (Table 1). Early or direct signaling events can thereby be distinguished from secondary signaling events resulting from autocrine signaling and negative feedback loops when analyzing the Boolean model. All logical equations with their assigned timescale constant are listed in S3 and S4 Tables. The housekeeping node, indicated by green boxes in the model scheme (S1 Fig), represents constitutively expressed genes, such as adaptor proteins and receptors that are already present in the unstimulated state of the cell. Thus, these nodes are activated at the first timescale τ = 0. The MyD88-dependent signaling events downstream of TLR4 leading to early NF-κB and MAP kinases activation as well as the signaling events downstream of the other receptor complexes are assigned to the timescale τ = 1. The MyD88-independent signaling pathway downstream of TLR4 accounting for late phase NF-κB activation is triggered at timescale τ = 2. All transcriptional and translational events take place at the timescale τ = 5 and τ = 7, respectively. In order to induce the autocrine feedback, the interferons as well as IL-1β, IL-6, and TNFα first need to be secreted by macrophages. Thus, these proteins occur twice in the model, one being the newly synthesized, intracellular protein at the bottom of the model scheme and one being the secreted protein at the top of the scheme activating the receptor on the cell surface. This secretion step is assigned to the timescale τ = 10. For synthesis and secretion of IL-10, first Stat3 needs to be activated by secretion of type I interferons and activation of IFNAR. Hence, IL-10 secretion occurs on a later timescale τ = 12. All inhibitory reactions are assigned to the last timescale τ = 15 to allow discrimination of feedforward signaling events induced by stimulation of macrophages and negative feedback loops for model analysis.

**Table 1:** Timescales of the Boolean model.

timescale τ	events
0	housekeeping
1	early TLR4 signaling (MyD88-dependent)
2	late TLR4 signaling (MyD88-independent)
5	transcription
7	translation
10	secretion of IL-1β, IL-6, TNFα, IFNα, IFNβ, IFNγ
12	secretion of IL-10
15	feedback inhibition

### Model analysis and validation

**Fig 1:**
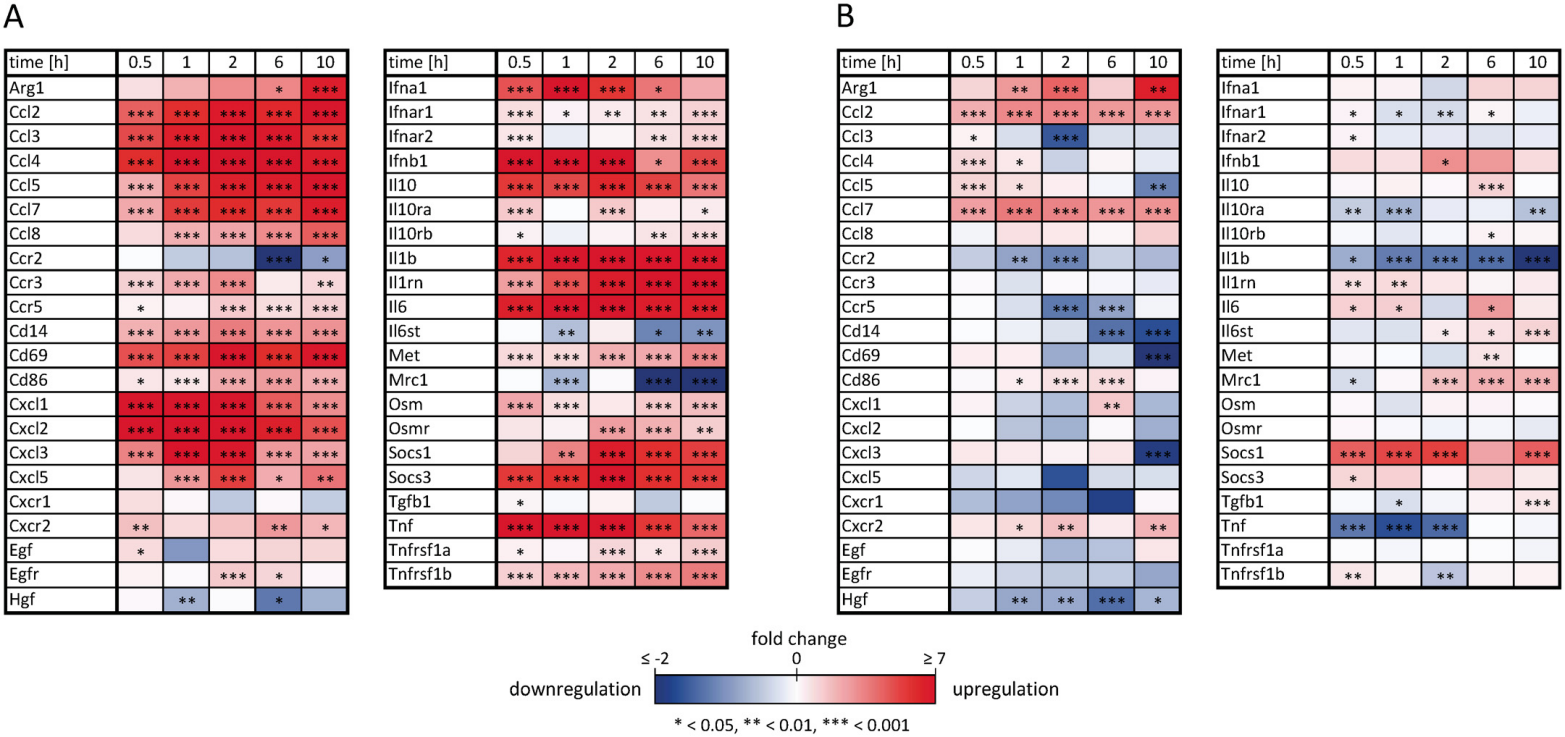
Inflammatory gene expression patterns of activated macrophages. Receptor and mediator transcript expression pattern of (A) M1 macrophages stimulated with LPS and (B) M2 macrophages stimulated with the interleukins IL-4 and IL-13 for 0.5, 1, 2, 6, and 10 measured using the high-throughput Taqman Fluidigm system. Data is analyzed using multivariable statistics and normalized to untreated controls. Genes marked in red and blue represent upregulated and downregulated genes, respectively (* *p* ≤ 0.05, ** *p* ≤ 0.01, *** *p* ≤ 0.001). For mean expression values with standard deviation, number of samples and p-values for regulation compared to untreated controls we refer to S1 Dataset.

For model validation, primary murine BMDMs are differentiated via macrophage colony stimulating factor (M-CSF) stimulation according to experimental guidelines suggested recently for the investigation of macrophage polarization [6]. LPS stimulation was used to induce M1 polarization whereas the M2 phenotype was triggered via stimulation with IL-4 and IL-13 for 0.5, 1, 2, 6 or 10 h. Prior to stimulation and after stimulation, macrophages were extensively characterized using cytometric analysis for the expression of different surface markers including CD14, F4/80, CD11b, CD68, CD69, CD86 and CD206 (S4 Fig). Quantitative, time-resolved data on 48 transcript expression levels were generated using the high-throughput Taqman Fluidigm Technology. Data were analyzed using multivariable regression [48], normalized to untreated controls and results are displayed as a heat map in Fig 1. The mean expression values with standard deviation, number of samples and p-values for regulation compared to untreated controls as well as the official full name and gene ID as given in the NCBI gene database and the Applied Biosystems assay number is presented in S1 Dataset.

**Fig 2:**
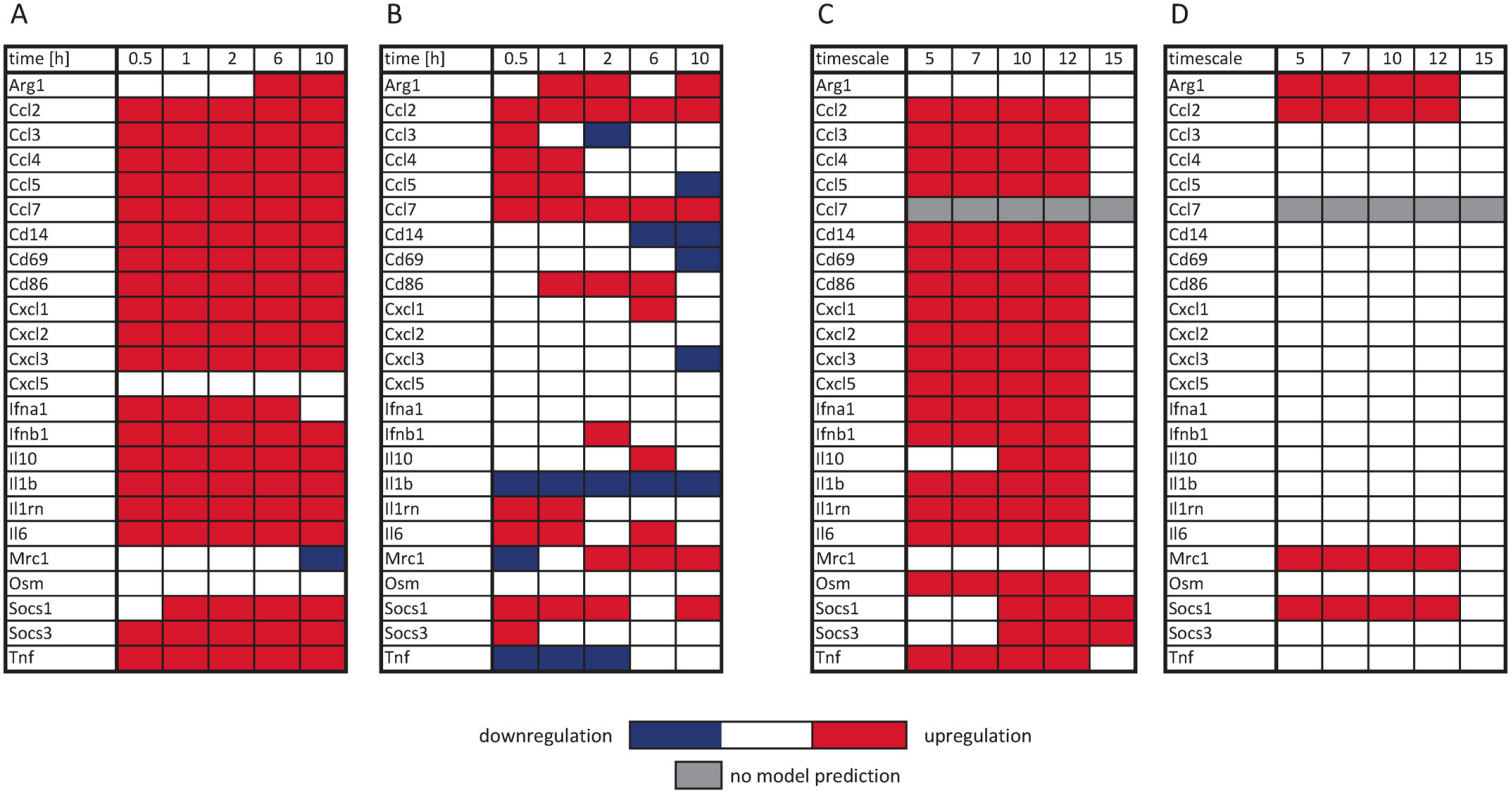
Comparison of experimental data and Boolean simulation results. Experimental data of (A) M1 and (B) M2 macrophages stimulated for 0.5, 1, 2, 6, and 10 h with LPS and IL-4/13, respectively. Quantitative data on mRNA expression is transformed into balanced ternary values, that is 1/-1 if the gene is significantly up-/downregulated at least 2-fold and 0 otherwise. Logical steady states of the mRNA species of the Boolean model for the input (C) LPS = 1, IL-4 = 0, IL-13 = 0 and (D) LPS = 0, IL-4 = 1, IL-13 = 1 for the time scales τ = {5, 7, 10, 12, 15}. The LSS for the timescales τ = {0, 1, 2} is not displayed since the first transcriptional step occurs not until τ = 5.

Among the 48 selected genes, we identified those clearly regulated above a threshold in response to either LPS or IL-4/13 stimulation (S1 Protocol 1.2) resulting in 16 and 7 genes that are regulated in M1 and M2 macrophages, respectively. In order to compare the Boolean model with time resolved data, the model was analyzed by calculating the logical steady states (LSS) [49] for a given input setting and timescale to study the resulting gene expression pattern of M1 vs. M2 macrophages at different time points. For better comparability, the quantitative data on mRNA expression were transformed into balanced ternary values that is 1/-1 if the gene is significantly up/downregulated at least 2-fold and 0 otherwise. Boolean simulation results and experimental data are compared in Fig 2. All pro-inflammatory chemokines and cytokines that are classified as upregulated from the measurement data of M1 macrophages (Fig 2A) are also upregulated in the Boolean model after LPS stimulation (Fig 2C). M2 macrophages, as already mentioned, are less well characterized compared to M1 macrophages. Thus, only four Stat6 target genes that are supposed to be regulated upon stimulation with IL-4/13 are part of the Boolean model (Fig 2D). Generally, M2 macrophages show a diminished response in the expression pattern of the analyzed genes compared to M1 macrophages (Fig 1), but upregulation of these four genes is confirmed by the experimental data (Fig 2B).

**Fig 3:**
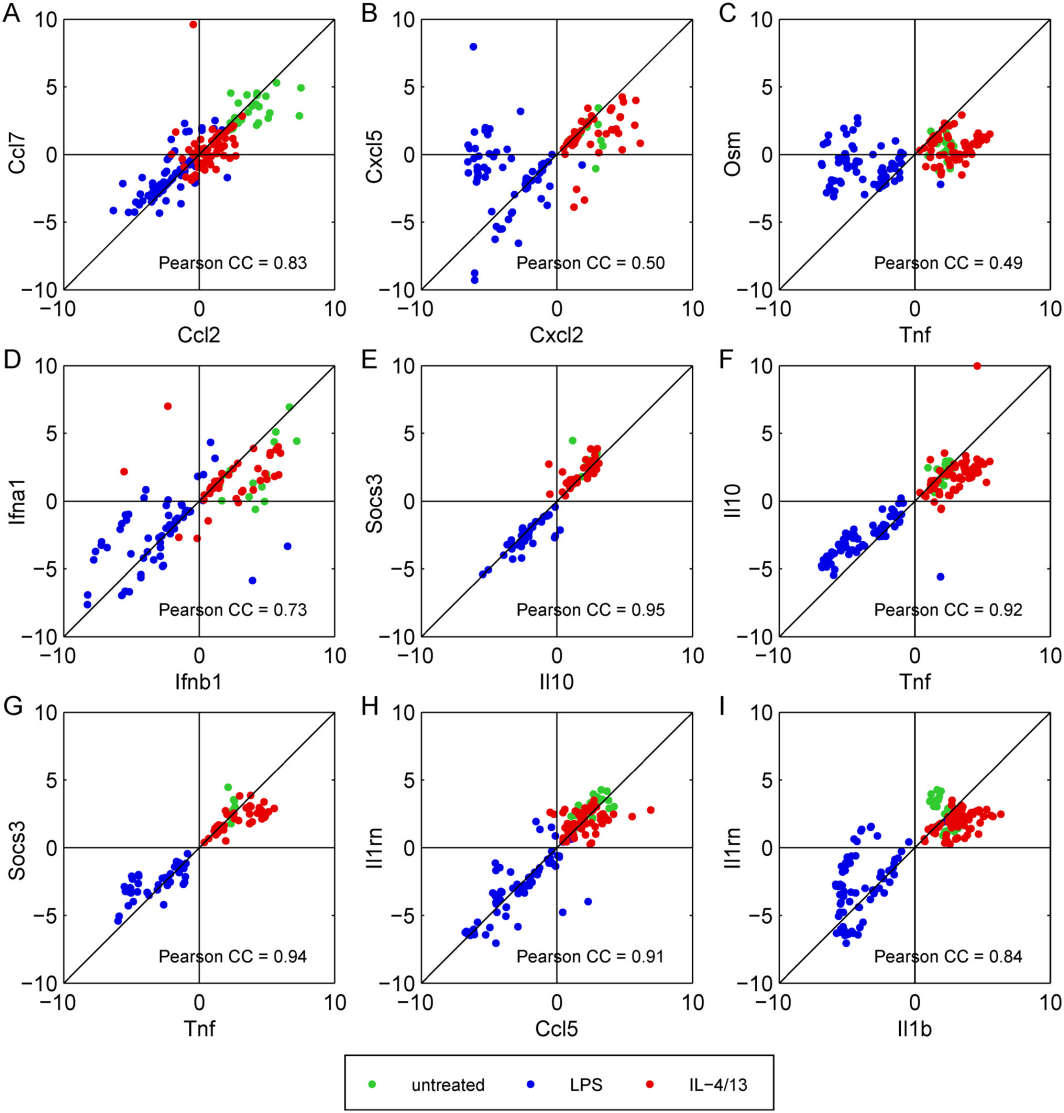
Co-expressed genes in macrophages. The scatter plots (A)-(I) indicate co-expression of the respective two genes if the Pearson’s correlation coefficient (CC) given is close to 1. For each gene, CT values minus mean expression value is shown.

However, there are some discrepancies between the measured and simulated expression patterns of M1 and M2 macrophages: (1) the chemokine Ccl7 is not part of the first model version but was shown to be upregulated in M1 and M2 macrophages (Fig 2A/2B) and co-expressed with Ccl2 (Fig 3A). Thus, Ccl7 was included in the model as NF-κB and Stat6 target gene in response to LPS and IL-4/13 stimulation, respectively. (2) The chemokine Cxcl5 was not upregulated at 0.5 h after LPS stimulation in contrast to the other CXC-type chemokines (Fig 2A). Furthermore, Cxcl-5 was not co-expressed with the other CXC-type chemokines such as Cxcl-2 (Fig 3B). Thus, Cxcl-5 as NF-κB target gene was removed from the model. (3) Similarly, Osm was not significantly regulated after LPS treatment (Fig 2A) and not co-expressed with typical NF-κB target genes such as TNFα (Fig 3C) and, therefore, was removed from the model. (4) Also, regulation of the type I interferon IFNα was less clear. It was not ranked as significantly upregulated (S1 Protocol 1.2) and was not clearly co-expressed with the other type I interferon IFNβ (Fig 3D) as predicted by the Boolean model and thus was also not considered in the model. (5) Likewise, IFNγ was not significantly expressed; values were close to or even beyond detection limit in most of the measurements. Hence, IFNγ must be excluded from analysis and was removed from the Boolean model. Since IFNγ was not expressed upon LPS and IL-4/13 stimulation, also IFNGR and downstream signaling events were removed. (6) The Boolean model predicts that IL-10 and Socs3 expression occurs later compared to the pro-inflammatory cytokines at τ = 10 (Fig 2C) since its expression requires secretion of type I interferons as well as activation of IFNAR. This is in contrast to the experimental results demonstrating that IL-10 and Socs3 were both similarly upregulated already 0.5 h after LPS stimulation comparable to the classical pro-inflammatory cytokines and such as TNFα (Fig 2A). Furthermore, IL-10 and Socs3 were shown to be co-expressed with TNFα (Fig 3F/3G). Since the transcription factor for early upregulation of IL-10 and Socs3 is still unknown but regulation of Socs3 expression via the p38^MAPK^/MK2 pathways has been shown [59, 65], early upregulation of these genes was included in the model in response to MK2 activation.

**Fig 4:**
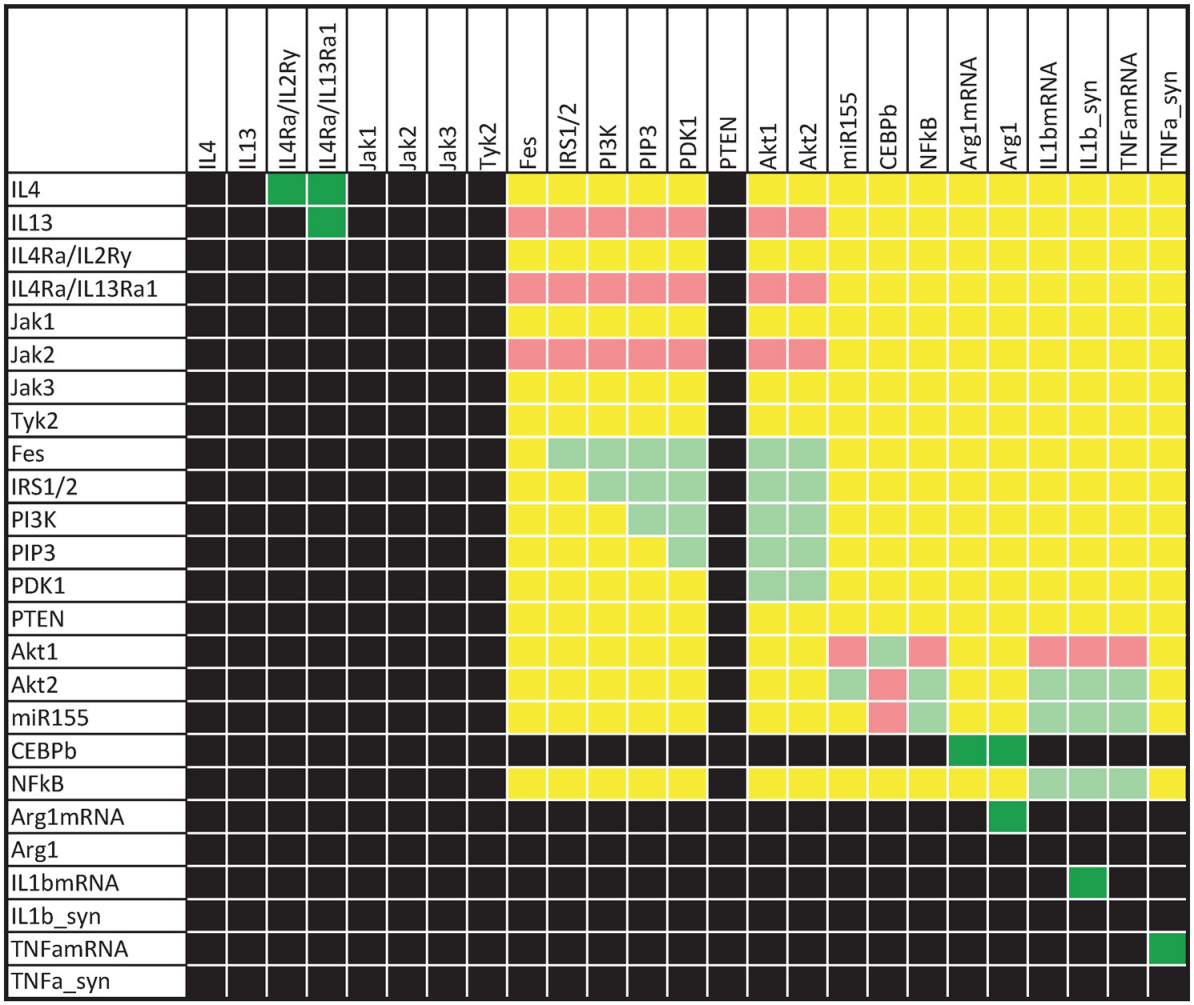
Dependency of IL-1β and TNFα expression on the PI3K/Akt pathway. The dependency matrix was calculated for the latest timescale (τ = 15). Each element *D*_*ij*_ of the matrix indicates the influence of species i on species j: black: no influence, yellow: ambivalent factor (both positive and negative paths connecting i with j), dark green/red: total activator/inhibitor (only positive/negative paths connecting i with j), light green/red: non-total activator/inhibitor (positive/negative paths connecting i with j, but at least one species is part of a negative feedback loop).

Downregulation is not represented by the Boolean simulation results (Fig 2C/2D). Nevertheless, the dependency matrix which covers the functional relation of all pairs of species in the Boolean model demonstrates an inhibitory influence of Akt1, which is the Akt isoform that mediates the primary effects in M2 macrophages after IL-4/13 stimulation, on IL-1β and TNFα expression (Fig 4, row 15, column 22–24). The influence of Akt1 on TNFα protein synthesis (TNFa_syn) is ambivalent (Fig 4, row 15, column 25), since Akt1 positively influences NF-κB activity and, thus, also favors expression of the phosphatase DUSP1, deactivation of p38 and inhibition of TNFα protein synthesis via TTP. Only the relevant section of the dependency matrix, that is the PI3K/Akt pathway, is presented for clarity.

### Dynamic modeling of the gene expression module

For a better understanding of the underlying dynamics of the system, particularly to investigate the time course of IL-10 and Socs3 upregulation that is differentially reflected by the Boolean model and the measurement data, the gene expression module is modeled dynamically using a system of ordinary differential equations (ODEs). To reduce complexity, the signaling paths are neglected and only mRNA expression as well as transcription factor activity as input *u*(*t*) of the system was modeled as explained in [52]. Briefly, information from the Boolean model regarding activated transcription factors and target genes of the respective pathways as well as the chronology of events is translated into five different input functions for the ODE model representing (*u*_1_) genes that are directly regulated via NF-κB upon LPS stimulation, (*u*_2_) genes that are regulated via NF-κB and secondary by the TFs Stat1 and Stat3 via autocrine feedback loops, (*u*_3_) genes, that are solely regulated via Stat TFs in response to LPS treatment, (*u*_4_) genes that are Stat6-dependently upregulated in response to IL-4/13 stimulation and (*u*_5_) genes that are repressed upon IL-4/13 stimulation. Expression of mRNA is given by

$$\frac{dX(t)}{dt}=k_{b}+k_{s}*u\left(t\right)-k_{d}*X\left(t\right)$$ (1)

where X denotes the relative mRNA concentration, *u*(*t*) the input function representing transcription factor activity (TFA) and *k*_*b*_, *k*_*s*_ and *k*_*d*_ the basal synthesis, induced synthesis and degradation rate constant, respectively. At *t* = 0, the system is at steady state, it is *u*(0) = 0 and, thus, scaling reduces the number of parameters by one per gene (S1 Protocol 2.1). If a gene is regulated in response to LPS as well as IL-4/13 stimulation, individual synthesis rates due to regulation by distinct transcription factors but identic degradation rates are assumed. Thus, the overall model contains 23 ODEs and 47 parameters, whereof eight are the time points determining TFA and 39 are synthesis and degradation rate constants. All model equations and parameter values can be found in S1 Protocol. The ODE model was implemented using the MATLAB Toolbox PottersWheel [51].

**Fig 5:**
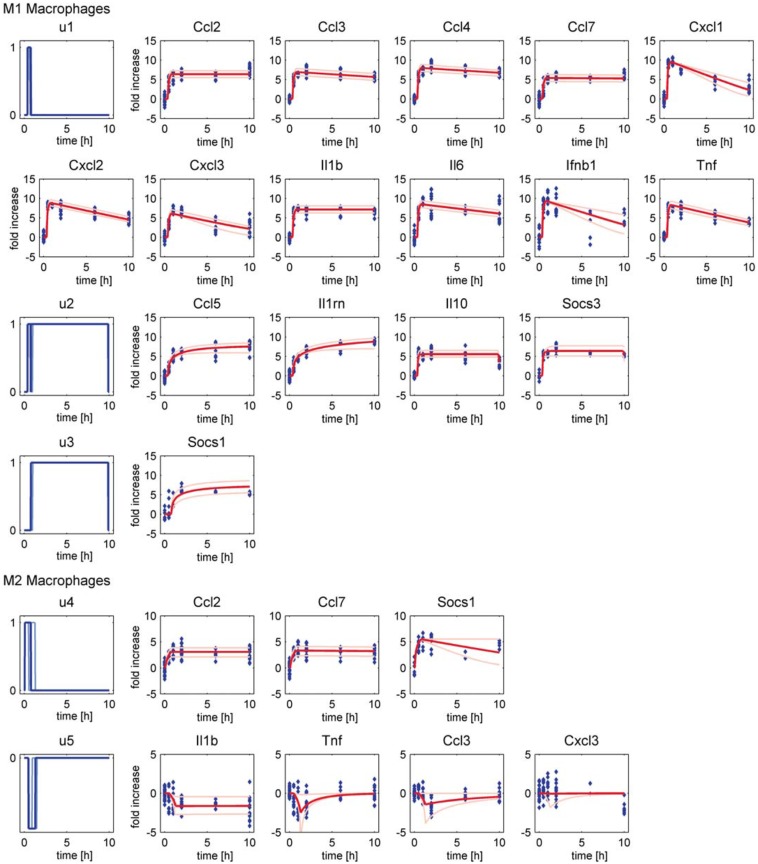
Simulation results of the ODE model describing mRNA expression in LPS- and IL-4/13-stimulated macrophages. Best fit of mRNA expression in LPS-stimulated M1 and IL-4/13-stimulated M2 macrophages is displayed as dark red line with the measurement points displayed as blue diamonds. The input functions u are displayed in the first column in dark blue and represent the regulation motifs: (*u*_1_) regulated by NF-κB, (*u*_2_) regulated by NF-κB and Stat3, (*u*_3_) regulated by Stat3, (*u*_4_) positively regulated after IL-4/13 stimulation by Stat6, and (*u*_5_) negatively regulated in response to IL-4/13. Bounds of the profile likelihood-based 68% confidence intervals are displayed in bright blue and red for input functions and mRNA expression, respectively.

Accordance of simulation results of the dynamic model and experimental data on transcript expression levels of murine BMDMs is shown in Fig 5 supporting our assumptions regarding transcriptional regulation of inflammatory mediators (1)–(6) mentioned above. Besides, one additional modification is implemented: (7) the IL-1 receptor antagonist (IL-1rn) was regulated similar to Ccl5 (Fig 1), that is both genes are slightly upregulated at 0.5 h after LPS stimulation but expression was increased at later time points. In contrast, the initial Boolean model predicted that IL-1rn is a NF-κB target gene such as IL-1β and thus upregulated only early after LPS stimulation. In fact, data analysis revealed that IL-1rn is co-expressed with Ccl-5 (Fig 3H) rather than with IL-1β (Fig 3I). Thus, IL-1rn was also included as Stat target gene similar to Ccl-5 in both the Boolean and the ODE model (*u*_2_).

**Fig 6:**
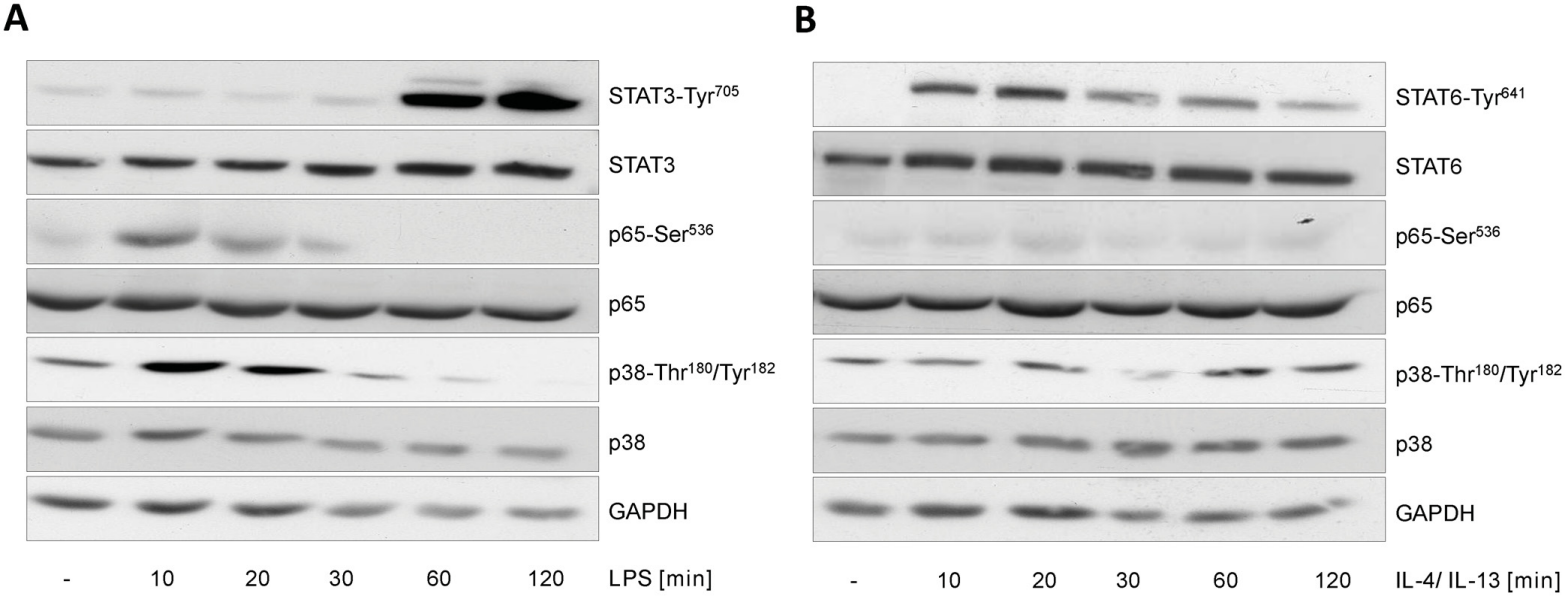
Transcription factor activity in macrophages under M1 (+LPS) or M2 (+IL-4/IL-13) stimulating conditions. Primary murine BMDMs were stimulated with (A) LPS (50 ng/ml) or (B) IL-4/IL-13 (25 ng/ml each) for the times indicated and whole cellular protein extracts were prepared. 30 μg of protein/lane were subjected to immunoblot analysis using antibodies specific for (A) STAT3-Tyr^705^, STAT3, p65-Ser^536^, p65, p38-Thr^180^/Tyr^182^, p38 or for (B) STAT6-Tyr^641^, STAT6, p65-Ser^536^, p65, p38-Thr^180^/Tyr^182^, p38 and GAPDH for loading control. Representative data for at least three independent experiments are shown.

**Fig 7:**
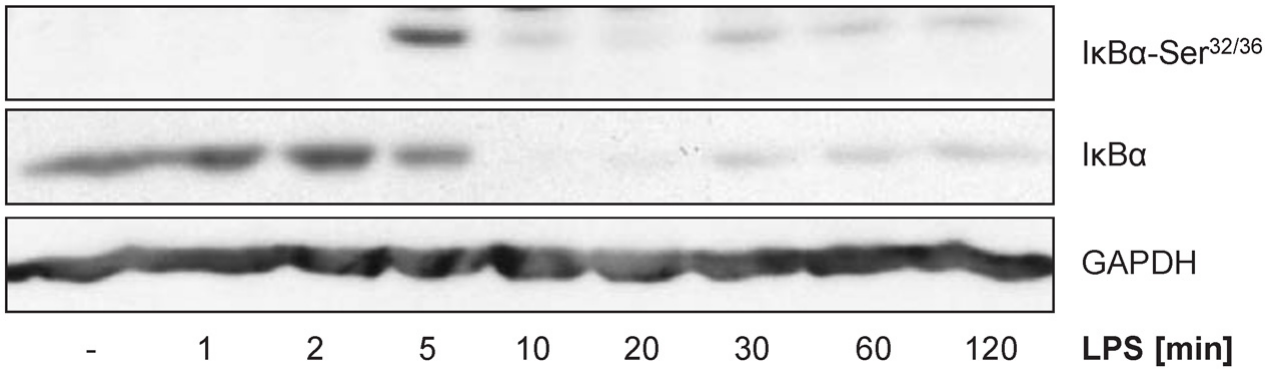
LPS dependent IκBα serine phosphorylation and degradation. Primary murine BMDMs were stimulated with LPS (50 ng/ml) for the times indicated. Whole cellular extracts were prepared. IκBα serine phosphorylation and IκBα protein expression was analyzed by Western Blot (60 μg of protein/lane). GAPDH served as loading control. Representative data for two independent experiments are shown.

**Fig 8:**
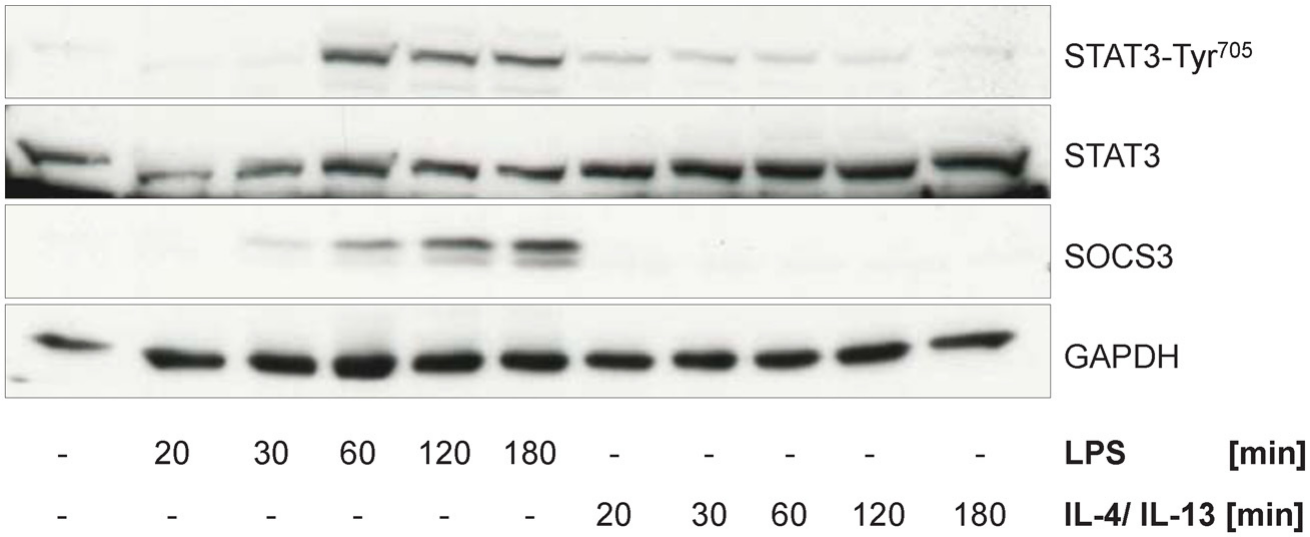
Jak/STAT signaling in response to LPS and IL-4/IL-13 stimulation. Primary murine BMDMs were stimulated with LPS (50 ng/ml) or IL-4/IL-13 (25 ng/ml each) for the times indicated and whole cellular protein extracts were prepared. 30 μg of protein/lane were subjected to immunoblot analysis using antibodies specific for STAT3-Tyr^705^, STAT3, SOCS3 and GAPDH. Representative data for at least three independent experiments are shown.

Identifiability of parameters was analyzed by investigating the profile likelihood using PottersWheel [53] and 68% confidence intervals were calculated (S1 Protocol 2.3). From the 47 parameters of the ODE model, 33 are identifiable and 14 parameters are practically non-identifiable (S5 Fig). But in most instances, the unidentifiability is caused by the quite low parameter values, for example eight non-identifiable parameters are degradation rate constants of relatively stable mRNAs that are not significantly degraded during the observed time frame. Thus, although there is some uncertainty in parameter values, variations of these parameters only has negligible influence on the model trajectories. Hence, the model is well suited to describe the important dynamics of mRNA regulation of the selected genes in M1 and M2 macrophages. Furthermore, it allows prediction of the time frame of TFA as described in [52]. Upon LPS stimulation, p65 is predicted to be active from 23 min to 46 min and Stat3 shortly afterwards from 47 min to 9.9 h. Following IL-4/13 stimulation, Stat6 is predicted to be active from 5 min to 47 min and transcriptional repression is predicted to be mediated from 30 min to 1.3 h after treatment. Transcription factor activity is confirmed by immunoblotting (Figs 6, 7 and 8).

### Secretion of cytokines and chemokines

**Fig 9:**
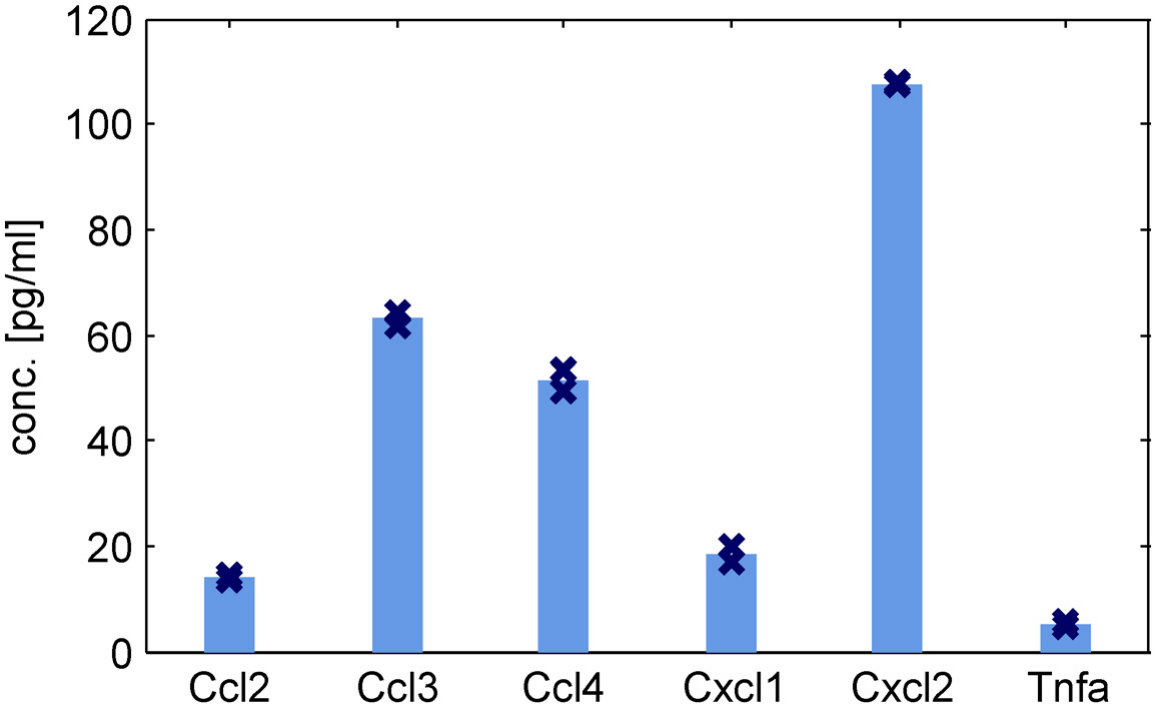
Proteins secreted by differentiated macrophages. Supernatant from macrophages (1 x 10^6^ cells) after 3 h subcultivation in 6 well plates was analyzed via Luminex Technology. Mean levels and individual data points (n = 2) are shown.

**Fig 10:**
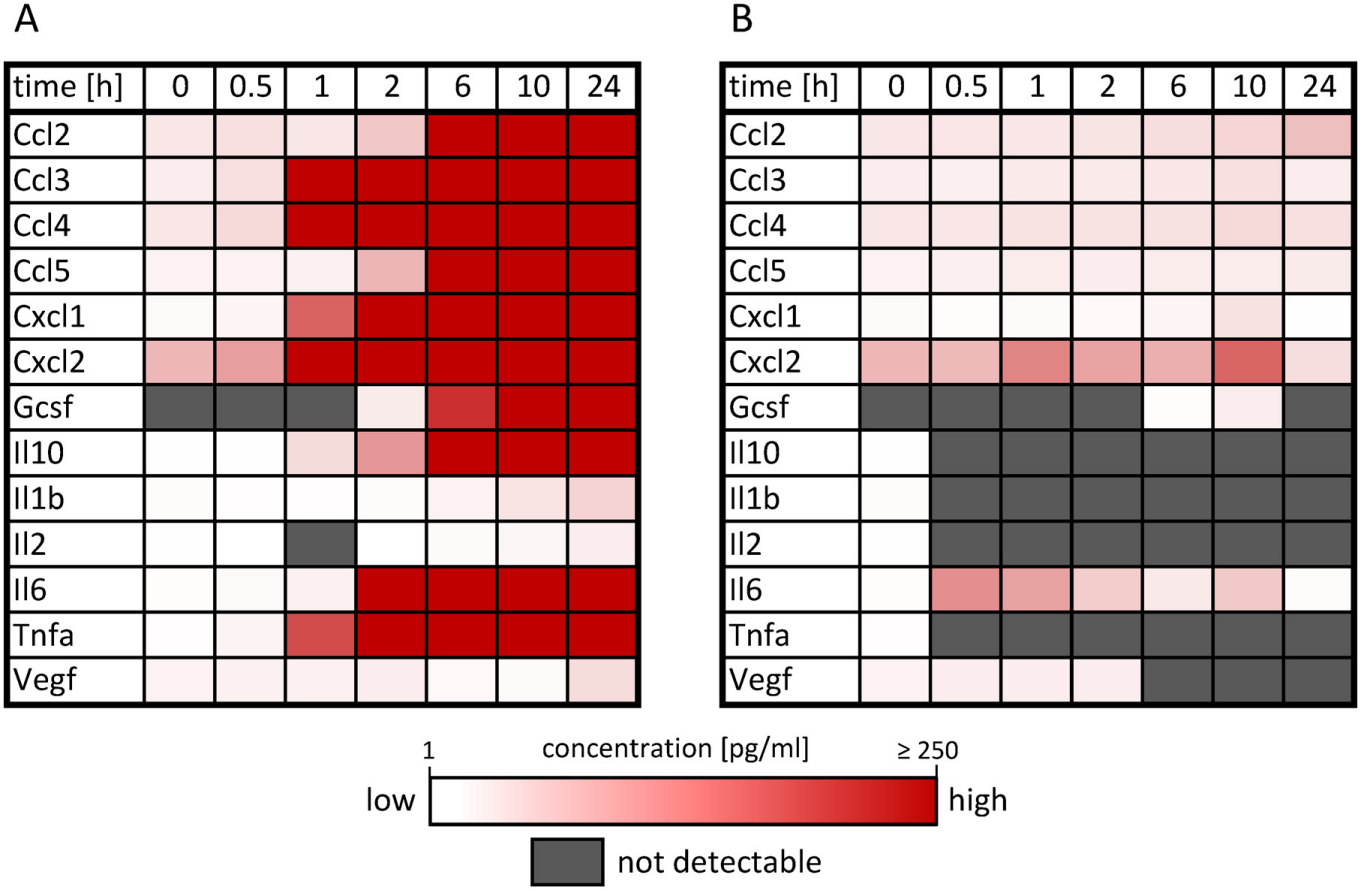
Protein secretion pattern of activated macrophages. Supernatant from macrophages ((1 x 10^6^ cells) treated with (A) LPS or (B) IL-4/13 analyzed via Luminex Technology at the indicated time points. For specific concentrations as well as standard deviation, number of samples and p-values for secretion compared to untreated controls we refer to S2 Dataset.

Upregulation of selected mediators secreted by macrophages was also confirmed on the protein level. Murine BMDMs were stimulated with LPS and IL-4/13 for 0.5, 1, 2, 6, 10 and 24 h and protein levels were quantified via Luminex Technology. Protein secretion of differentiated macrophages was also investigated. Macrophages were differentiated for eight days and then 1 x 10^6^ cells were subcultivated in 6 well plates, as usual. Supernatant from those cells was collected after 3 h of cultivation and analyzed via Luminex Technology. Differentiated macrophages constitutively secrete low amounts of the chemokines Ccl2, Ccl3, Ccl4, Cxcl1, and Cxl2 as well as TNFα (Fig 9). Significant increase of quantified cytokines and chemokines is detectable after 1 to 6 h after LPS stimulation (Fig 10A). Especially the chemokines are secreted in high concentration after LPS treatment: Ccl3, Ccl4, Cxcl1, and Cxcl2 are secreted after 1 h. Their concentration in the supernatant of cells reach 38.9, 35.1, 14.2 and 66.4 ng/ml after 24 h stimulation, respectively. The chemokines Ccl2 and Ccl5 increase after 6 h LPS stimulation and reach 3.5 and 9.4 ng/ml after 24 h. Significant increase of IL-6 is quantified after 2 h stimulation and 182.4 ng/ml are detectable after 24 h. TNFα increases after 1 h LPS stimulation and reaches 2.5 ng/ml after 24 h, whereas IL-10 reaches 0.45 ng/ml. IL-1β is secreted in lower concentration, 43.4 pg/ml are detectable after 24 h of LPS treatment. After IL-4/13 stimulation, the secretion of chemokines is only slightly increased compared to the unstimulated macrophages (Fig 10B). Interestingly, the cytokines IL-1β, IL-2, IL-10, and TNFα are not detectable in the supernatant of IL-4/13 stimulated macrophages. Secretion of Cxcl2 and IL-6 is transiently increasing. For specific concentrations we refer to S2 Dataset.

Further adaptions of the network topology of activated macrophages were performed after analysis of protein data: (8) Stimulation with LPS leads to IκBα phosphorylation (serine residues 32 and 36) and, subsequently, to the degradation of IκBα (Fig 7). Furthemore, LPS stimulation induces phosphorylation of p65 on serine 536, promoting the transactivation by NF-κB (p65/p50) [66, 67]. This only occurs in the first hour following LPS stimulation, as estimated using the dynamic model of mRNA expression (Fig 5) and confirmed via immunoblotting (Figs 6A and 7). On the other hand, first significant increase of the cytokines in the supernatant of cells was detectable only after one hour LPS stimulation (Fig 10A). Thus, no effect of these cytokines on NF-κB transactivating activity is visible and, thus, the autocrine feedback loops of IL-1β and TNFα are removed from the Boolean model. (9) Similarly, when IL-6 is secreted about 2 h after LPS stimulation, Stat3 is already activated (Figs 6A and 8) and Socs3 is highly upregulated on the mRNA (Figs 1 and 5) and also on the protein level (Fig 8) inhibiting Stat phosphorylation at IL-6R and IFNAR. Only IL-10 is able to mediate late and sustained Stat3 activation, since the IL-10R is insensitive to Socs3 [60]. The autocrine feedback loop of IL-6 resulting in Stat3 activation is therefore removed from the model.

### Model refinement

**Fig 11:**
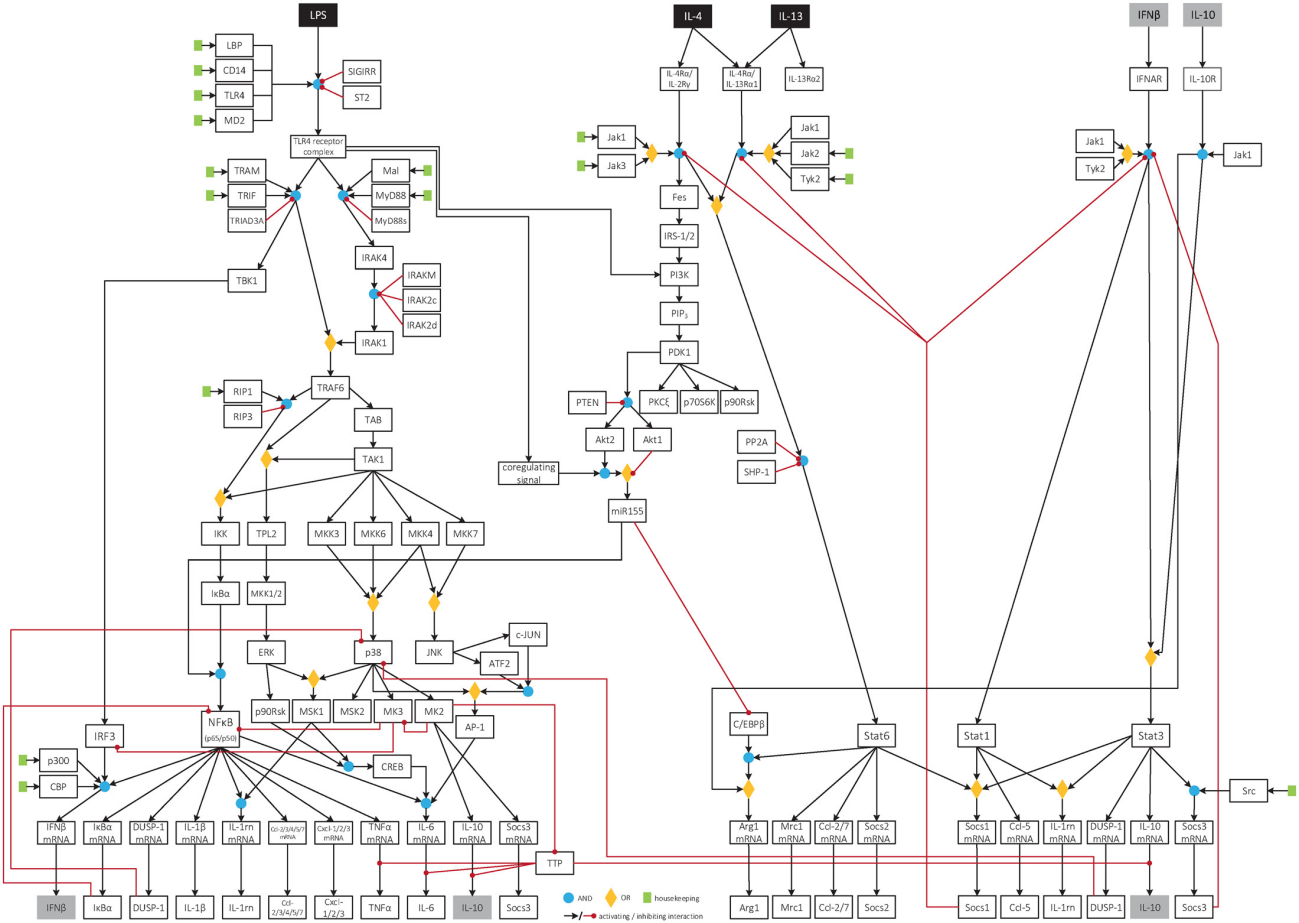
Refined version of the Boolean model of M1 and M2 macrophage activation. This model describing macrophage activation into the M1 and M2 phenotype is specifically adapted to measurement data from our experimental system of primary murine BMDMs. Inputs of the system are LPS, IL-4 and IL-13 depicted in black. The model is displayed as logical interaction hypergraph containing 132 nodes and 152 interactions. Black arrows and red lines denote activating and inhibiting interactions, respectively. Logical AND connections are expressed using blue dots, and OR connections by yellow diamonds. The housekeeping node is depicted by green rectangles indicating all species that are already present in the unstimulated state of the cell. Grey-shaded species denote cytokines that are secreted by the cells and are able to mediate autocrine feedback.

The combination of modeling and experimental analysis of the inflammatory genes expressed in activated macrophages resulted in the refined version of the Boolean model as presented in Fig 11. The assumptions mentioned in the previous section (1)–(9) are implemented in the refined version of the Boolean model that is now specifically adapted to our experimental system of primary murine BMDMs. Compared to the first literature-based version (S1 Fig), 13 interactions were modified, 33 were deleted and 9 interactions were newly introduced to the Boolean model. Accordingly, 22 species were deleted from the first model, 6 species were added and 12 were modified in the final model version. The final model contains 132 nodes and 152 interactions. All abbreviations and description of network nodes as well as the logic equations are given in S1 and S3 Tables. Modified entries are indicated and the deleted nodes and interactions are listed in S2 and S4 Tables. The model now contains two autocrine feedback loops mediated by secreted IFNβ and IL-10 indicated by grey boxes.

## Discussion

In this study, we present a comprehensive characterization of M1 (LPS-activated) and M2 (IL-4/13-activated) macrophages and their specific cytokine and chemokines expression profile. We combined current knowledge about macrophages across several cell types and species in the first version of the Boolean model. Based on established procedures [6, 7, 44, 68, 69], a protocol for the generation of primary murine BMDMs and their activation into well characterized M1(LPS) and M2(IL-4/13)-type macrophages was generated. Furthermore, the Boolean model was validated and refined with expression data of cytokines and chemokines on the mRNA and partially also on the protein level.

The simulation results of the first Boolean model were compared to mRNA data of BMDMs resulting in elimination of some genes that were not significantly regulated, such as the type II interferon IFNγ whose expression is already controversially reported in literature. Originally, it was assumed that IFNγ production is restricted to activated T cells and natural killer cells, but it has been shown that murine BMDMs secrete large amounts of IFNγ in response to IL-12 and IL-18 stimulation [70]. Another group showed production of IFNγ mRNA and protein in murine macrophages in response to LPS stimulation without detectable amounts of secreted protein [71]. Recently, it has been shown that murine peritoneal macrophages produce moderate amounts of IFNγ in response to LPS stimulation [72]. However, for our system of murine BMDMs, IFNγ was neither detectable on the mRNA nor the protein level in response to stimulation with LPS and IL-4/13. Also, the type I interferon IFNα was removed since it was not clearly upregulated in murine BMDMs in contrast to IFNβ indicating that IFNβ is one of the principal type I interferons triggering IL-10 dependent sustained activation of STAT3 in response to LPS via an autocrine or paracrine feedback loop.

Expression of interferons after TLR4 activation is regulated by two TFs, NF-κB and IRF3, the first is activated via the early MyD88-dependent pathway and the latter via the delayed MyD88-independent pathway [11]. These two signaling pathways were hence modeled on two separate time scales in the Boolean model. However, no difference in onset of transcription was visible when examining the mRNA data, probably because the measurement time points were too sparse and at the first time point of 0.5 h already both TFs were activated. Furthermore, there is evidence that although IRF3 is activated later than NF-κB, both TFs translocate to the nucleus together to activate transcription [73]. Thus, there is no need to include IRF3 and distinct regulation of IFNβ mRNA in the dynamic model of mRNA expression and we focused on NF-κB as major transcription factor directly activated in LPS-stimulated macrophages. After analyzing the time course of mRNA regulation in macrophages in more detail using the ODE model of gene expression, we found that the IL-1 receptor antagonist IL-1rn is not only regulated by NF-κB but also at later time points possibly via the Stat TFs. IL-1rn was already described as a Stat6 target gene [16]. Besides, it was shown that inhibition of NF-κB via adenoviral transfection of IκBα decreases expression of IL-1rn only marginally [74] and that IL-1rn is an IL-10 inducible gene [75]. Also IL-10 and Socs3 turned out to be regulated earlier before its cognate TF Stat3 is activated as shown by the dynamic ODE model and confirmed by immunoblot analysis (Figs 6A and 8). Regulation of IL-10 expression is diversely reported in literature. Some state that expression of IL-10 is NF-κB independent [74, 76]. Saraiva et al. showed that a NF-κB p65/RelA site was activated in the IL-10 promotor after stimulation of primary mouse BMDMs through pattern recognition receptors [77]. Also Staples and colleagues demonstrated that IL-10 regulates IL-10 expression itself by an autocrine feedback loop via the TF Stat3, but the initial IL-10 expression following LPS stimulation is Stat3-independent [78]. So there is a high probability that NF-κB is involved in initial regulation of IL-10 expression, but also the MAP kinases p38^MAPK^ and ERK as well as the p38^MAPK^ downstream effector kinase MAPK activated protein kinase 2 (MK2) were shown to play a role [17, 79, 80].

The final modification was removing the autocrine feedback loops of IL-1β, TNFα and IL-6. Since these cytokines are the major ones inducing the acute phase response in hepatocytes [15, 36], it is plausible that they primarily act on surrounding cell types such as hepatocytes and to a negligible amount back on the macrophages. This is supported by the dynamical model of mRNA expression. Although there are some uncertainties in the parameters, the model is well suited to determine the important dynamics of mRNA regulation as well as the time of transcription factor activity which was confirmed by immunoblot analysis (Figs 6 and 8). Only 14 of 47 parameters are practically non-identifiable. Thereof, eight parameters are mRNA degradation rate constants of IL-1β, IL-1rn, Socs1 and the CCL-type chemokines whose lower bounds are non-identifiable implying that mRNAs are relatively stable and not necessarily degraded over the observed time span of 10 h. Similarly, the lower bounds of the two synthesis rate constants of Ccl3 and Cxcl3 following IL-4/13 stimulation are non-identifiable. Although, by analyzing the measurement data these genes were determined to be downregulated it is also possible that they are non-regulated after stimulation with IL-4/13. The last four practically non-identifiable parameters are those determining transcription factor activity. More precisely, the lower bounds of the parameters describing the time point of Stat3 activation in LPS-stimulated macrophages and the time point for Stat6 activation as well as the time point for beginning of negative regulation following IL-4/13 stimulation are non-identifiable. This indicates that regulation in response to IL-4/13 is relatively quickly due to the very short signaling paths. For LPS-stimulated macrophages, this indicates that activity of NF-κB and Stat3 is possibly slightly overlapping. Furthermore, the upper bound of the parameter for the time point of Stat3 deactivation is non-identifiable indicating that Stat3 could be active longer than the observed time frame of 10 h after LPS stimulation.

In this study, we choose the phenotype of M1 or classically activated macrophages on the one side and alternatively activated or M2 macrophages on the other side for comparative analysis, since these two represent the initially described and most prominent phenotypes [7]. The M1 and M2 terminology originated from the correlating concept of the TH1 and TH2 dichotomy. Later, the concept of M1/M2 macrophage activation was extended as it became clear that activated macrophages are highly heterogeneous [8]. A concept has been evolved describing many shades of macrophage activation with the three main phenotypes, namely classically activated macrophages, wound-healing macrophages, and regulatory macrophages with mixed phenotypes in between, for example tumor-associated macrophages [1]. In a recent proposal, this concept has been refined and a nomenclature has been suggested that is linked to the activation standards [6]. Compared to this classification, the M2 macrophages activated by IL-4 and IL-13 as described in this study would be termed as M(IL-4/IL-13). As mentioned, classically activated M1(LPS) and wound-healing M2(IL-4/IL-13) macrophages represent two major phenotypes that are involved in a number of diseases that mainly result from a disturbed balance of macrophage activation with lacking restoration of homeostasis after injury. Thus, systematic characterization of the distinct macrophage phenotypes as well as the corresponding cytokine and chemokine expression patterns as represented by the final, refined version of the Boolean model is particularly important for improving the current understanding of macrophage activation and their roles in health and disease. In this context, the model provided suggests that miRNA-155 represents a critical molecule that determines how activation of Akt by the different stimuli is involved in the determination of the activation type of macrophages. We observed differential regulation of miRNA-155 expression following stimulation with either LPS or IL-4 and IL-13 which is in line with recent concepts demonstrating that the PI3K/Akt pathway plays a critical role in the control of macrophage plasticity with the two Akt isoforms Akt1 and Akt2 being important regulators of M2 and M1 differentiation, respectively [34, 35]. Among others this may involve Akt1 dependent phosphorylation of the downstream kinase GSK3β at serine 9, which inhibits GSK3β activity [81] thereby interfering with activation of p65/p50 NF-κB dimers by enhancing formation of the inhibitory p50/p50 NF-κB dimer [82]. Moreover, isolated activation of Akt1 in Akt2 deficient macrophages prevents expression of miRNA-155 resulting in an increased availability of CEBP/β that in turn mediates expression of target genes such as Arginase1, IL-10 and YM1. On the other hand isolated activation of Akt2 in Akt1 deficient macrophages leads to an enhanced activation of p65/p50 NF-κB dimers and subsequent upregulation of p65/p50 dependent gene expression [35]. In line with this study, we showed increased expression of miRNA-155 after LPS stimulation which induces M1 polarization and NF-κB-dependent upregulation of inflammatory mediators. Accordingly, stimulation with IL-4 and IL-13 led to decreased levels of miRNA-155. Notably, although they share with PI3K the same upstream activator, Akt1- and Akt2-driven pathways have distinct regulatory functions with respect to macrophages polarization. This makes it likely that the decision in which direction the polarization is driven is mediated by PI3K-independent co-regulatory signals which are integrated either at the level of the two Akt isoforms Akt1 or Akt2 or at the level of their downstream signaling. Everts et al. [83] recently described a similar role of TBK1 in the context of dendritic cell activation. They identified the kinase TBK1 downstream of the activated TLR4 receptor complex as most important kinase mediating Akt phosphorylation after LPS stimulation. The crosstalk of LPS and PI3K/Akt signaling in the context of macrophage activation needs further investigation.

## Materials and Methods

### Animals and ethic statement

8–12 weeks old male C57BL/6J mice were used for the generation of BMDMs. All animal applications were reviewed and approved by the appropriate authorities and were performed in accordance with the German animal protection law (Landesamt für Natur, Umwelt und Verbraucherschutz Nordrhein-Westfalen, Recklinghausen. Animals were handled and housed according to specific pathogen free (SPF) conditions in the local breeding facility (ZETT, Zentrale Einrichtung für Tierforschung und Tierschutzaufgaben, Heinrich-Heine-University Düsseldorf).

### Preparation and cultivation of primary murine bone marrow derived macrophages (BMDMs)

Macrophages derived from bone marrow cultures were obtained according to the method of Meerpohl et al. [44] with some modifications. In brief, both femurs and tibiae of male, 8–12 weeks old C57BL/6J mice were removed and dissected free of adherent tissue. Intact bones were kept in sterile phosphate buffered saline (PBS; Biochrom, Berlin, Germany). Before starting bone marrow preparation, bones were left in 70% [v/v] ethanol for 3–5 minutes, washed with PBS and transferred into washing medium (DMEM 1000 mg/ml Glucose; Biochrom, Berlin, Germany supplemented with 1% [v/v] Penicillin/Streptomycin; PAN Biotech, Aidenbach, Germany). After cutting off the ends of the bones, the bone marrow was flushed out by irrigation with washing medium using syringe and needle (23G x 1“). A single cell suspension was obtained by pipetting cells vigorously followed by centrifugation (1200 rpm, 4°C, 10 minutes, low acceleration/ deceleration). Cells were resuspended in culture medium (DMEM 1000 mg/ml Glucose, 1% [v/v] Penicillin/Streptomycin, 10% [v/v] fetal calf serum) without murine macrophage colony stimulating factor (M-CSF). The viable cell counts were performed in a hemocytometer using trypan blue solution. The cell viability was at least about 95–97% and 54 x 10^6^ ± 3.8 cells/mouse (mean ± SEM) were isolated. Cells were seeded in three vented cell culture flasks (75 cm^2^) for overnight incubation at humidified atmosphere (37°C, 5% CO_2_). Adherent cells (e.g. stromal fibroblasts) will settle out. The next day, the non-adherent cells were harvested for further cultivation by performing centrifugation. Cells were resuspended in culture medium, supplemented with 10 ng/ml M-CSF (Peprotech, Rocky Hill, USA). Again, trypan blue exclusion was performed and total cell amount was calculated: 37 x 10^6^ ± 2.0 cells/mouse (mean ± SEM). Cells were seeded for proliferation and macrophage differentiation in five cell culture dishes with 15 cm diameter and 20 ml medium (+M-CSF) each [45]. 10 ml of medium supplemented with M-CSF was added on day 3, 6 and 7 (no aspiration of media). After 8 days of cultivation, adherent cells were harvested by gentle trypsinisation: Cells were washed twice with prewarmed PBS and treated with 3 ml trypsin/EDTA (PAN Biotech, Aidenbach, Germany) for approximately 5–10 minutes. Cells were centrifuged and after evaluation of the total cell yield (39 x 10^6^ ± 2.7 cells/mouse (mean ± SEM)), they were adjusted in M-CSF containing culture medium: 3 x 10^6^ cells/3 ml/6 well plate cavity for RNA isolation and 1 x 10^6^ cells/2 ml/6 well plate cavity for supernatant collection. Medium was changed to FCS free culture medium 6 hours before performing the experiments. Cells were stimulated with 50 ng/ml LPS or 25 ng/ml IL-4/ IL-13, respectively. RNA was isolated or cell supernatant was collected after 0.5, 1, 2, 6 and 10 hours of stimulation.

### RNA isolation, cDNA synthesis and qRT-PCR

Total RNA was isolated using the RNeasy Miniprep Kit (Qiagen, Hilden) according to manufacturer’s instructions. Assessment of RNA integrity and quantity was performed by using Agilent Technology (Agilent 2100 Bioanalyzer, Waldbronn, Germany). 600 ng total RNA was reverse transcribed to cDNA with TaqMan Reverse Transcription Reagents (Applera GmbH, Darmstadt, Germany). For qRT-PCR we used the Fluidigm’s BioMark HD high-throughput quantitative chip platform (Fluidigm Corporation, San Francisco, CA, USA) with pre-designed gene expression assays from Applied Biosystems according to the manufacturer’s instructions [46]. All TaqMan assays are listed in S1 Dataset.

### miRNA isolation and qRT-PCR

For analysis of miRNA expression cells were grown in 6 well plates and treated with 50 ng/ml LPS, with 25 ng/ml IL-4/IL-13 each or left untreated. Total cellular RNA including miRNA was isolated using the miRNeasy Miniprep Kit from Qiagen (Hilden, Germany) according to the manufacturer’s instructions. 100 ng of total RNA was reverse transcribed with miScript II Reverse Transcription Kit (Qiagen, Germany) using the HiFlex buffer for transcription of mature and precursor miRNA as well as ncRNA and mRNA. cDNA was diluted 1:5, and 2 μl of the diluted cDNA was added as template to a final volume of 25 μl including 1 x QuantiTect SYBR Green PCR master mix according to the manufacturer’s instructions (Qiagen, Germany). The following primers were used for real time PCR: mouse miRNA-155 (sense, 5’-TTA ATG CTA ATT GTG ATA GGG GT-3’; antisense, miScript universal primer) and the mouse U6 snRNA (sense, 5’-CGC TTC GGC AGC ACA TAT AC-3’; antisense, 5’-AAA TAT GGA ACG CTT CAC GA-3’). Except the universal primer, which was from Qiagen, Germany, all oligonucleotides were purchased from Eurofins MWG Operon (Ebersberg, Germany). No-template and no-reverse-transcriptase controls were used to control specificity of RT-PCR. Semi-quantitative PCR results were obtained using the ddCT method. Expression values of the miRNA were normalized to the expression levels of the control gene U6 snRNA and referred to untreated controls. Data from four independent experiments are presented as mean ± standard error of the mean (SEM).

### Antibodies, reagents, cell culture materials

Antibodies for cytometric analysis were purchased from ebioscience: CD14 (#12-0141-81), CD11b (#12-0112-81), F4/80 (#17-4801-80) from BD Pharmingen: CD69 (# 553237), CD86 (558703) from Dianova: CD68 (#MA1-82739) and AbD Serotec: CD206 (#MCA2235FB). For exclusion of background fluorescence, cells were stained with the respective isotype control antibodies in parallel: Rat IgG2b K Isotype Control PE, Armenian Hamster IgG Isotype Control PE, Rat IgG2b K Isotype Control APC, Rat IgG2a K Isotype Control FITC (ebioscience). Anti-p65-Ser^536^, anti-STAT3-Tyr^705^, anti-p38-Thr^180^/Tyr^182^, anti-p38, anti-IκBα-Ser^32/36^, anti-IκBα and anti-STAT6-Tyr^641^ antibodies used for Western Bot analysis were obtained from Cell Signaling Technology (Berverly, MA, USA). Anti-STAT3, anti-p65 and anti-STAT6 antibodies were purchased from Santa Cruz Biotechnology (Santa Cruz, CA, USA), the antibody against SOCS3 was obtained from IBL (Minneapolis, MN, USA) and against GAPDH from Biodesign (Saco, ME, USA). Cell culture reagents as Dulbecco’s modified Eagle medium (DMEM) were purchased from Biochrom (Berlin, Germany), FCS (Cat.: 10099141, Lot: 769367) was obtained from Invitrogen (Karlsruhe, Germany), Penicillin G/ Streptomycin and Trypsin/ EDTA-Solution were from Cytogen (Wetzlar, Germany). Recombinant murine M-CSF, IL-4 and IL-13 were obtained from Peprotech (Rocky Hill, NJ, USA). LPS from E. coli (#L3012) was purchased from Sigma-Aldrich (München, Germany).

### Protein isolation and western immunoblotting

The protocol for total protein isolation, Western Immunoblotting and signal detection has been described previously [47]. Equal amounts of protein were subjected to SDS/PAGE. The electrophoretically separated proteins were transferred to polyvinylidene difluoride (PVDF) membranes by the semidry Western blotting method. The immunoblots were developed with the enhanced chemiluminescence system (Amersham Biosciences, MA, USA) following the manufacturer’s instructions.

### Quantitative cytokine measurement

At the end of the experimental treatment, cell culture supernatant was collected under sterile conditions followed by centrifugation (20 minutes, 4°C, 5.500 rpm). Aliquots were pre-cooled at -20°C and stored in -80°C until quantitative mediator measurement. Analysis of cytokine concentration in supernatants was performed by using Luminex Technology (Austin, TX, USA) and the MCYTOMAG-70K Mouse Cytokine/Chemokine Magnetic Bead Panel from Millipore (Billerica, MA, USA) according to manufacturer’s instruction. Cell culture media and untreated control samples were used for background normalization.

### Cytometric analysis

Maturation of macrophages and characterization of macrophage polarization was assessed by using fluorescence-activated cell sorting (FACS) and was performed using the FACS Canto II (BD Biosciences, Heidelberg, Germany). Briefly, after differentiation, cells grown in 6 well cavities were harvested by trypsinization (3 minutes, 37°C). Reaction was stopped by adding 1 ml FACS buffer (PBS w/o Ca^2+^/Mg^2+^, 2% FCS, 2 mM EDTA, 0.1% NaN_3_). After centrifugation, fixation and permeabilization was performed using the Leucoperm Reagent Kit (AbD Serotec, Kidlington, UK) followed by antibody treatment according to manufacturer’s instructions. Each experiment was performed in at least three independent cell preparations using the same experimental conditions. Data analysis was performed with FlowJo Software, version 7.6.5 (Ashland, OR, USA).

### Statistical analysis

Values are expressed as means ± standard deviation, where indicated. Fluidigm data was analyzed using LEMming, a multivariable statistics approach [48] and normalized to untreated controls. Outlier were discarded as explained in S1 Protocol 1.1. Differential expression was assessed using the two-sample Student’s t-tests. All computations were conducted using RStudio Version 0.98.1091 and MATLAB R2014a.

### Boolean modeling

The Boolean model is presented as logical interaction hypergraph (LIH) as introduced by Klamt et al. [49], that is two or more species participate in one interaction. Each species is defined by a logical, binary state variable, that is 0/1 for inactive/active state or absent/present. The model was implemented using the MATLAB Toolbox CellNetAnalyzer (CNA) [50]. Logical AND connections are represented by blue dots in the LIH, OR connections by yellow and inhibiting interactions are indicated by red lines. A so called timescale constant τ [49] is assigned to each logical interaction indicating when a reaction becomes active, whereat reactions with lower timescale constants become active earlier than interactions with higher timescale constants. Abbreviations and descriptions of species are given in S1 and S2 Tables, logical equations can be found in S3 and S4 Tables. S1 Model contains all necessary files for starting the Boolean model of macrophage activation with the CellNetAnalyzer.

### Boolean model analysis: calculation of logical steady states (LSS)

The input/output behavior of the Boolean model is analyzed by calculating the LSS for a given input setting, that is LPS = 1, IL-4 = 0, IL-13 = 0 for M1 macrophages and LPS = 0, IL-4 = 1, IL-13 = 1 for M2 macrophages. During LSS analysis, the signal is propagated from the fixed inputs along the logical hyperarcs as far as uniquely feasible till the system reaches a LSS, that is the state of each species is consistent with its assigned Boolean function. Assignment of time scales does not modify the manner of LSS calculation rather than altering the analyzed Boolean network by extracting smaller subnetworks. Calculating a LSS for a time scale τ = *t* computes the LSS for a subnetwork containing all interactions with a time scale constant τ ≤ *t* whereas interactions with a time scale constant τ > *t* are not included in the analysis [49].

### Dynamic modeling

The gene expression model is based on ordinary differential equations (ODEs) and mass action kinetics. It was implemented using the MATLAB Toolbox PottersWheel [51] and comprises 23 species and 47 parameters. The model setup is explained in [52], a short summary is given in S1 Protocol as well as a list of model equations and parameter values. The ODE system is numerically integrated using the Sundials solver CVODES and parameter values were estimated by least squares fitting. Identifiability of parameters was analyzed by estimating the profile likelihood as explained in [53].

## Supporting Information

S1 Fig First version of the Boolean model describing macrophage activation. Literature-based Boolean model describing the response of macrophages to the inputs LPS, IL-4 and IL-13 depicted in black representing the phenotype of classically activated (LPS-stimulated, M1 phenotype) as well as alternatively activated (IL-4/13 stimulated, M2 phenotype) macrophages. The model is displayed as logical interaction hypergraph containing 148 nodes and 176 interactions. Black arrows and red lines denote activating and inhibiting interactions, respectively. Logical AND connections are expressed using blue dots and OR connections by yellow diamonds. The housekeeping node is depicted by green rectangles indicating all species that are already present in the unstimulated state of the cell. Grey-shaded species denote cytokines that are secreted by the cell and are able to mediate autocrine feedback.
(TIFF)

S2 Fig Influence of Akt1 and Akt2 on macrophage polarization in the Boolean model. The first version of the Boolean model predicted (A) M1 polarization after LPS stimulation and (B) also M1 polarization after IL-4 stimulation. In order to restore correct M2 polarization after IL-4 stimulation an additional coregulating signal is necessary that is induced by the activated TLR4 receptor complex and, together with Akt1, enhances miRNA-155 expression. In the corrected version of the Boolean model (C) M1 polarization is induced by LPS and (D) M2 polarization is induced by IL-4, as expected.
(TIF)

S3 Fig LPS and IL-4/IL-13 dependent expression of miRNA-155. Primary murine BMDMs were incubated with 50 ng/ml LPS or with 25 ng/ml of IL-4/IL-13 each. Following, the expression of miRNA-155 was determined as described in the Materials and Methods section at the indicated time points. Data were analysed using the ddCT method and mRNA levels are depicted as fold of untreated controls. Data are presented as mean ± SEM (n = 4). Significances are indicated by * for *p* ≤ 0.05, ** for *p* ≤ 0.01 and *** for *p* ≤ 0.001.
(TIF)

S4 Fig Macrophage marker expression determined by flow cytometry analysis. Macrophages were treated with LPS (50 ng/ml) or IL-4/ IL-13 (25 ng/ml each) as indicated and cells were subjected to FACS analysis, as described in Materials and Methods. (A) CD14, F4/80, CD68 and CD11b expression, (B) CD69 and CD86 expression and (C) CD206 expression after one day of cultivation. Arithmetic means of histogram GeoMean (fold of control) ± SEM of at least three independent experiments are shown.
(TIF)

S5 Fig Profile likelihood versus all parameters of the ODE model of mRNA expression. Profile likelihood is displayed by blue lines, estimated parameter values are indicated by green circles. The threshold for 68% confidence intervals is depicted as red line. Parameter values on the base 10 logarithmic x-axis are displayed in orders of magnitude. The χ^2^_PL_ value is calculated by the sum of mean square errors. For calculation of each profile likelihood, the respective parameter value is decreased and increased based on the estimated optimal value in various steps whereas all other parameter values are again optimized by weighted least-square minimization. If the profile likelihood crosses the threshold line, the parameter is considered as identifiable. Otherwise, the upper or lower bound of the parameter remains unidentifiable.
(TIF)

S1 Table Nodes of the Boolean model of macrophage activation. Notation of all species of the Boolean model (Fig 11) with their official full name and gene ID according to the NCBI gene data base or description. An asterisk (*) in the last column indicates nodes that are newly added after comparison of experimental data and simulation results of the first version of the Boolean model (S1 Fig). A hash (#) indicates a modified node; the corresponding species in S2 Table is also marked with a hash.
(PDF)

S2 Table Deleted or modified nodes. List of all species that were part of the first literature-based version of the Boolean model (S1 Fig) but were modified or deleted after comparison with experimental data and thus do not occur in the final model version (S1 Table). A hash (#) indicates a modified node; the corresponding species in S1 Table is also marked with a hash.
(PDF)

S3 Table Interaction equations of the Boolean model of macrophage activation. List of all logical equations of the Boolean model (Fig 11) in the notation of the CellNetAnalyzer with their assigned timescale constant τ, a short description and reference. An asterisk (*) in the last column indicates equations that are newly added after comparison of experimental data and simulation results of the first version of the Boolean model (S1 Fig). A hash (#) indicates a modified equation; the corresponding interaction equation in S4 Table is also marked with a hash.
(PDF)

S4 Table Deleted or modified interaction equations. List of all logical equations that were part of the first literature-based version of the Boolean model (S1 Fig) but were modified or deleted after comparison with experimental data and thus do not occur in the final model version (S3 Table). A hash (#) indicates a modified equation; the corresponding interaction in S3 Table is also marked with a hash.
(PDF)

S1 Protocol The file contains more detailed information on data analysis and modeling. (PDF)

S2 Protocol Boolean model of macrophage activation. The file contains the refined Boolean model of macrophage activation implemented with CellNetAnalyzer [50]. CNA is freely available for academic use. Software and online manual can be downloaded from http://www.mpi-magdeburg.mpg.de/projects/cna/cna.html. After starting CNA, a new signal-flow project has to be created using the provided folder ‘MacrophageActivation’. Textboxes are optimized for width 0.007, height 0.015, and font size 8.
(ZIP)

S1 Dataset Transcript expression data of activated macrophages. Mean expression values of measured mRNAs from primary murine BMDMs stimulated with LPS or IL-4/13 for 0.5, 1, 2, 6 and 10 h normalized on untreated controls with standard deviation, number of samples and p-values for regulation compared to untreated controls as well as the official full name and gene ID as given in the NCBI gene database and the Applied Biosystems assay number.
(XLSX)

S2 Dataset Protein secretion data of macrophages. Mean expression values of measured proteins in the supernatant of primary murine BMDMs after differentiation and stimulation with LPS or IL-4/13 for 0, 0.5, 1, 2, 6, 10 and 24 h with standard deviation, number of samples and p-values for secretion compared to untreated controls (*t* = 0).
(XLSX)
