# Supplementary figures and images for: Model-Based Characterization of Inflammatory Gene Expression Patterns of Activated Macrophages
Source: PLoS Comput Biol. 2016 Jul 27;12(7):e1005018. doi: 10.1371/journal.pcbi.1005018 (PMC4963125; doi:10.1371/journal.pcbi.1005018)

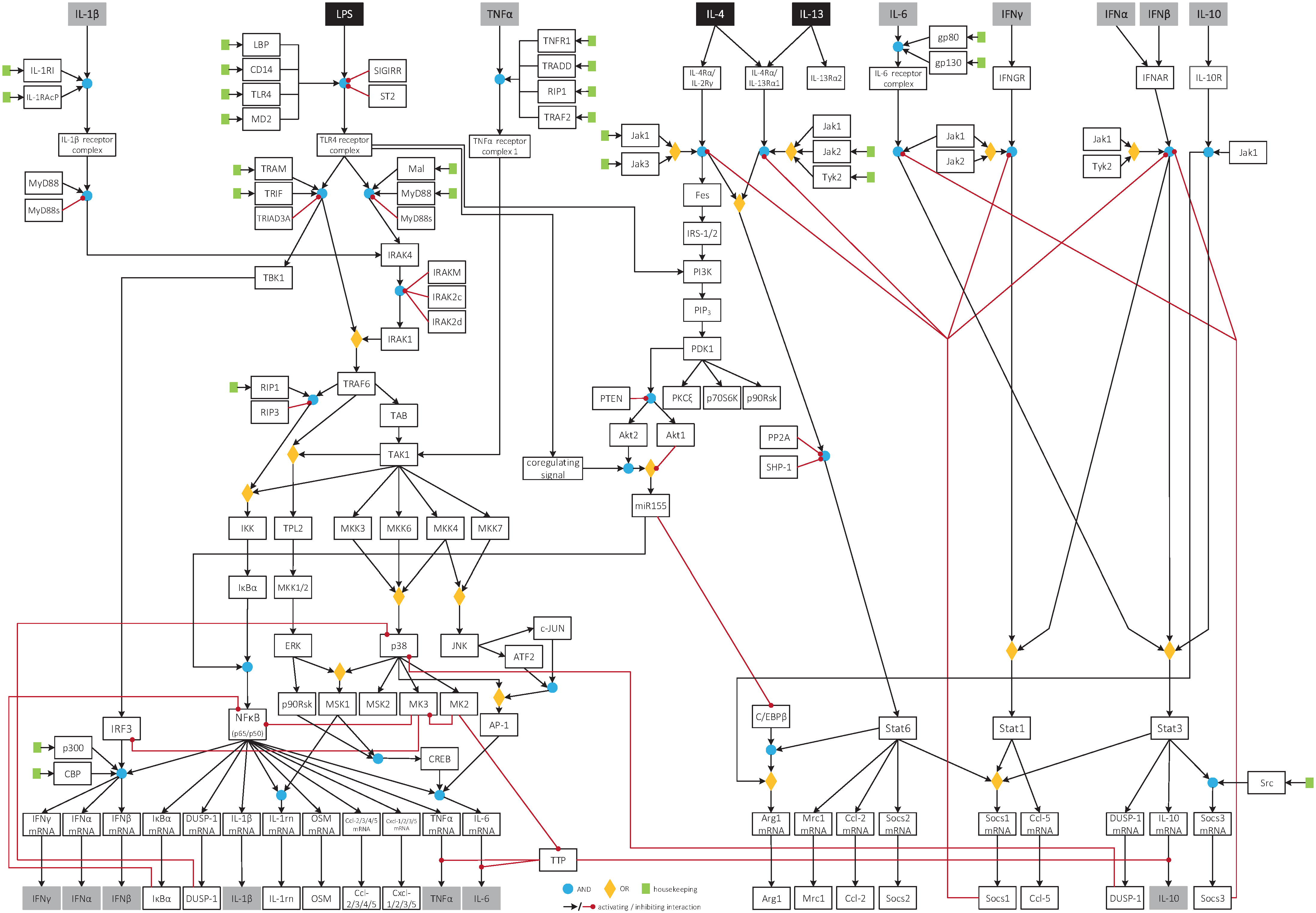

Supplement: S1 Fig — Literature-based Boolean model describing the response of macrophages to the inputs LPS, IL-4 and IL-13 depicted in black representing the phenotype of classically activated (LPS-stimulated, M1 phenotype) as well as alternatively activated (IL-4/13 stimulated, M2 phenotype) macrophages. The model is displayed as logical interaction hypergraph containing 148 nodes and 176 interactions. Black arrows and red lines denote activating and inhibiting interactions, respectively. Logical AND connections are expressed using blue dots and OR connections by yellow diamonds. The housekeeping node is depicted by green rectangles indicating all species that are already present in the unstimulated state of the cell. Grey-shaded species denote cytokines that are secreted by the cell and are able to mediate autocrine feedback. (TIFF) [file pcbi.1005018.s001.tiff]

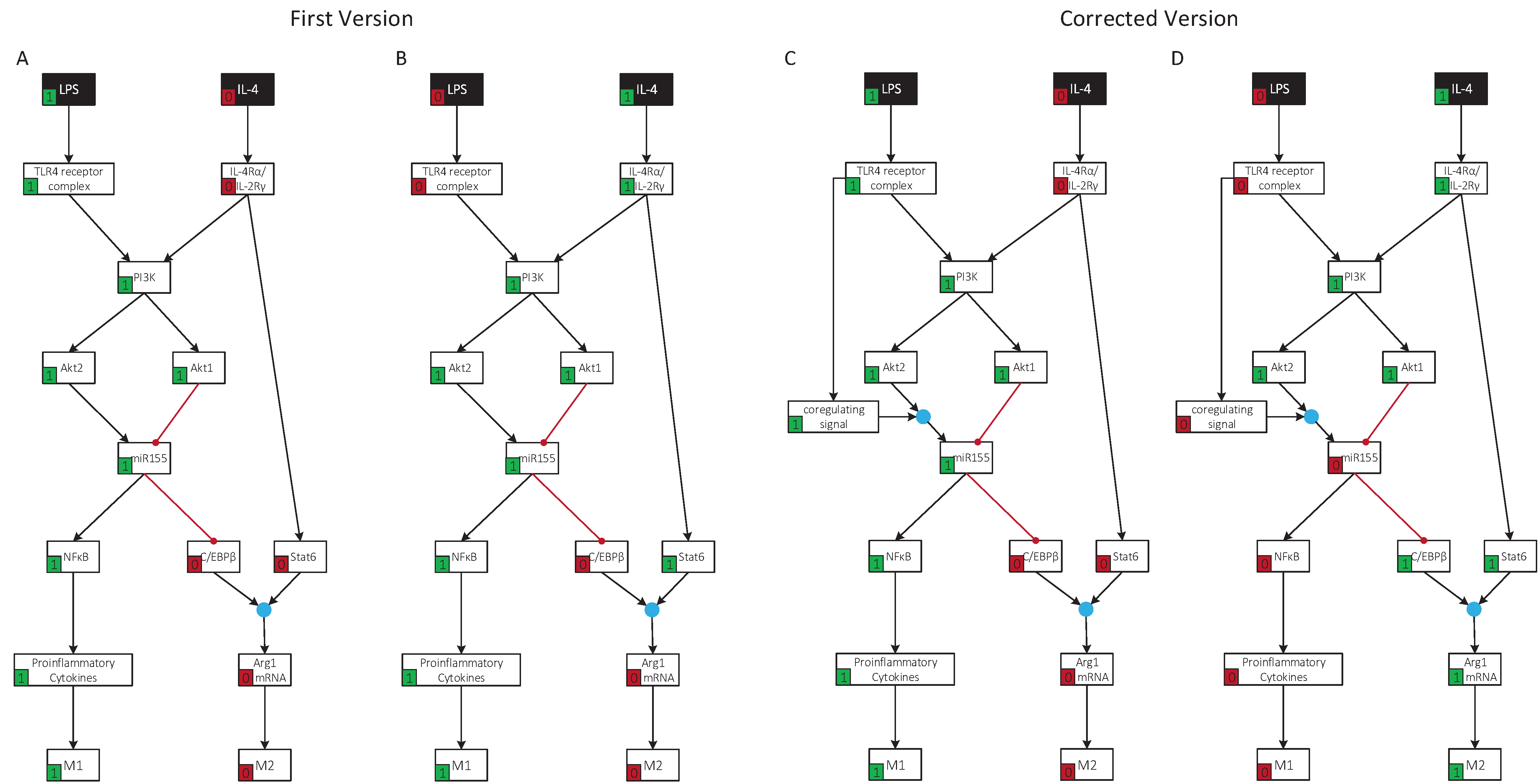

Supplement: S2 Fig — The first version of the Boolean model predicted (A) M1 polarization after LPS stimulation and (B) also M1 polarization after IL-4 stimulation. In order to restore correct M2 polarization after IL-4 stimulation an additional coregulating signal is necessary that is induced by the activated TLR4 receptor complex and, together with Akt1, enhances miRNA-155 expression. In the corrected version of the Boolean model (C) M1 polarization is induced by LPS and (D) M2 polarization is induced by IL-4, as expected. (TIF) [file pcbi.1005018.s002.tif]

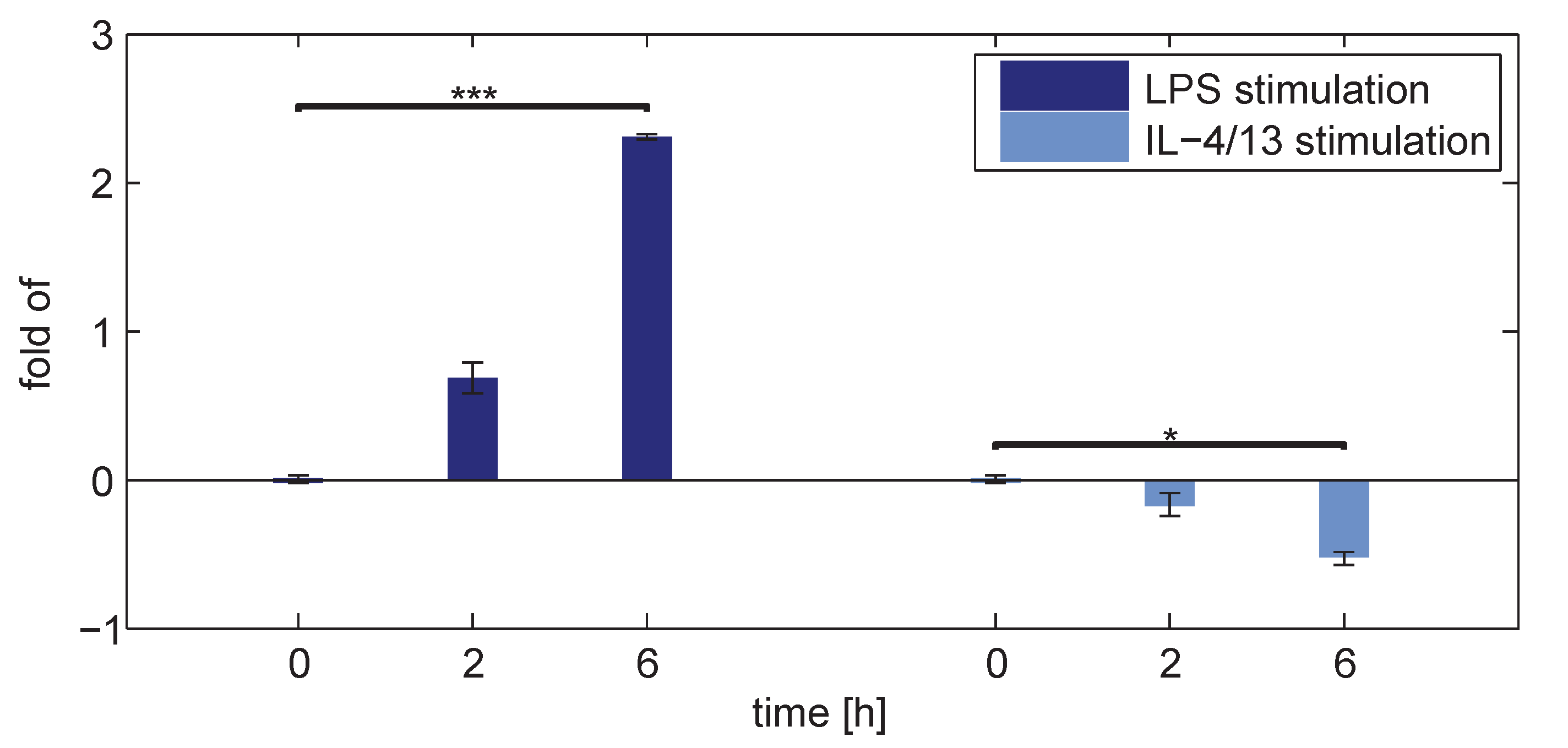

Supplement: S3 Fig — Primary murine BMDMs were incubated with 50 ng/ml LPS or with 25 ng/ml of IL-4/IL-13 each. Following, the expression of miRNA-155 was determined as described in the Materials and Methods section at the indicated time points. Data were analysed using the ddCT method and mRNA levels are depicted as fold of untreated controls. Data are presented as mean ± SEM (n = 4). Significances are indicated by * for p ≤ 0.05, ** for p ≤ 0.01 and *** for p ≤ 0.001. (TIF) [file pcbi.1005018.s003.tif]

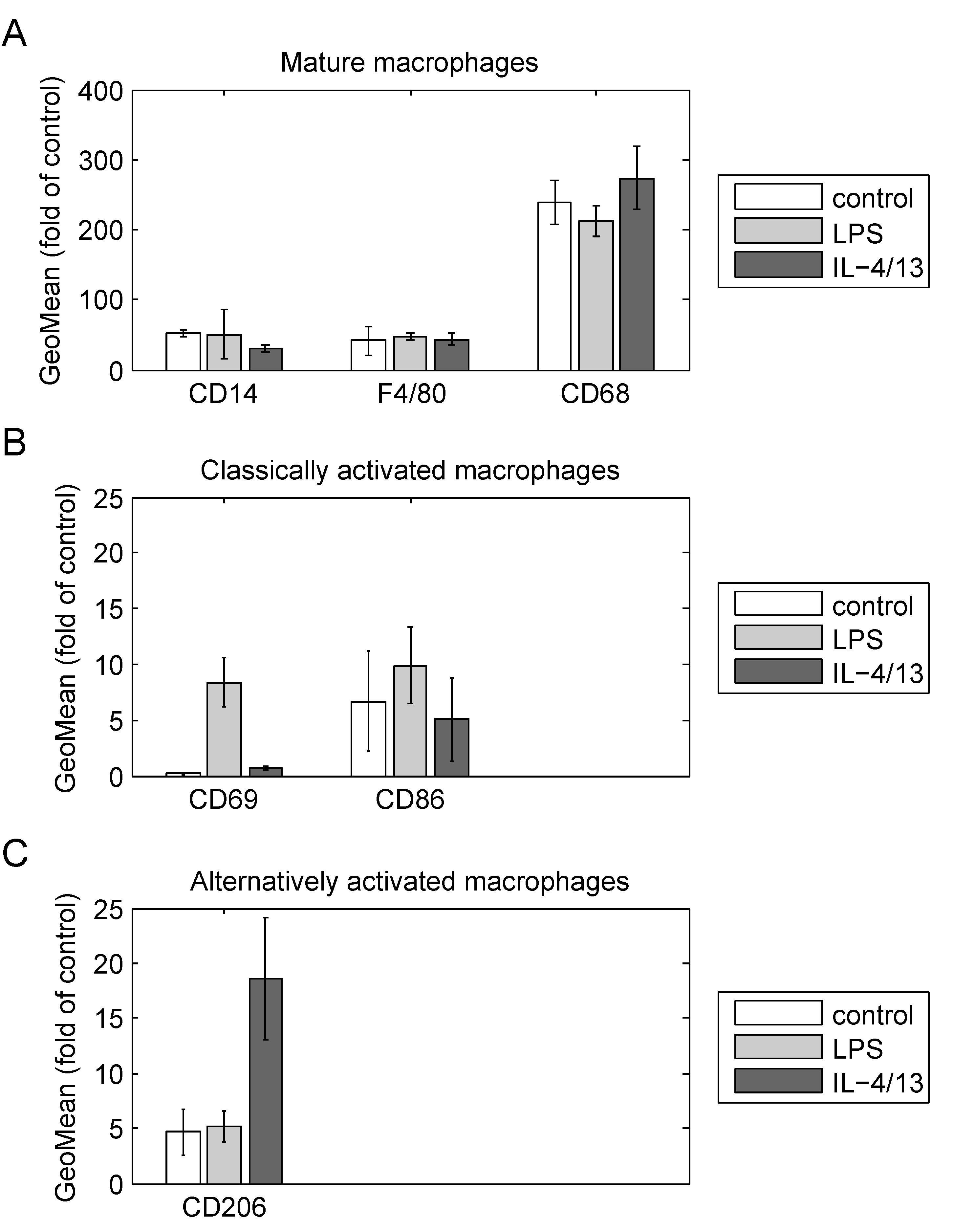

Supplement: S4 Fig — Macrophages were treated with LPS (50 ng/ml) or IL-4/ IL-13 (25 ng/ml each) as indicated and cells were subjected to FACS analysis, as described in Materials and Methods. (A) CD14, F4/80, CD68 and CD11b expression, (B) CD69 and CD86 expression and (C) CD206 expression after one day of cultivation. Arithmetic means of histogram GeoMean (fold of control) ± SEM of at least three independent experiments are shown. (TIF) [file pcbi.1005018.s004.tif]

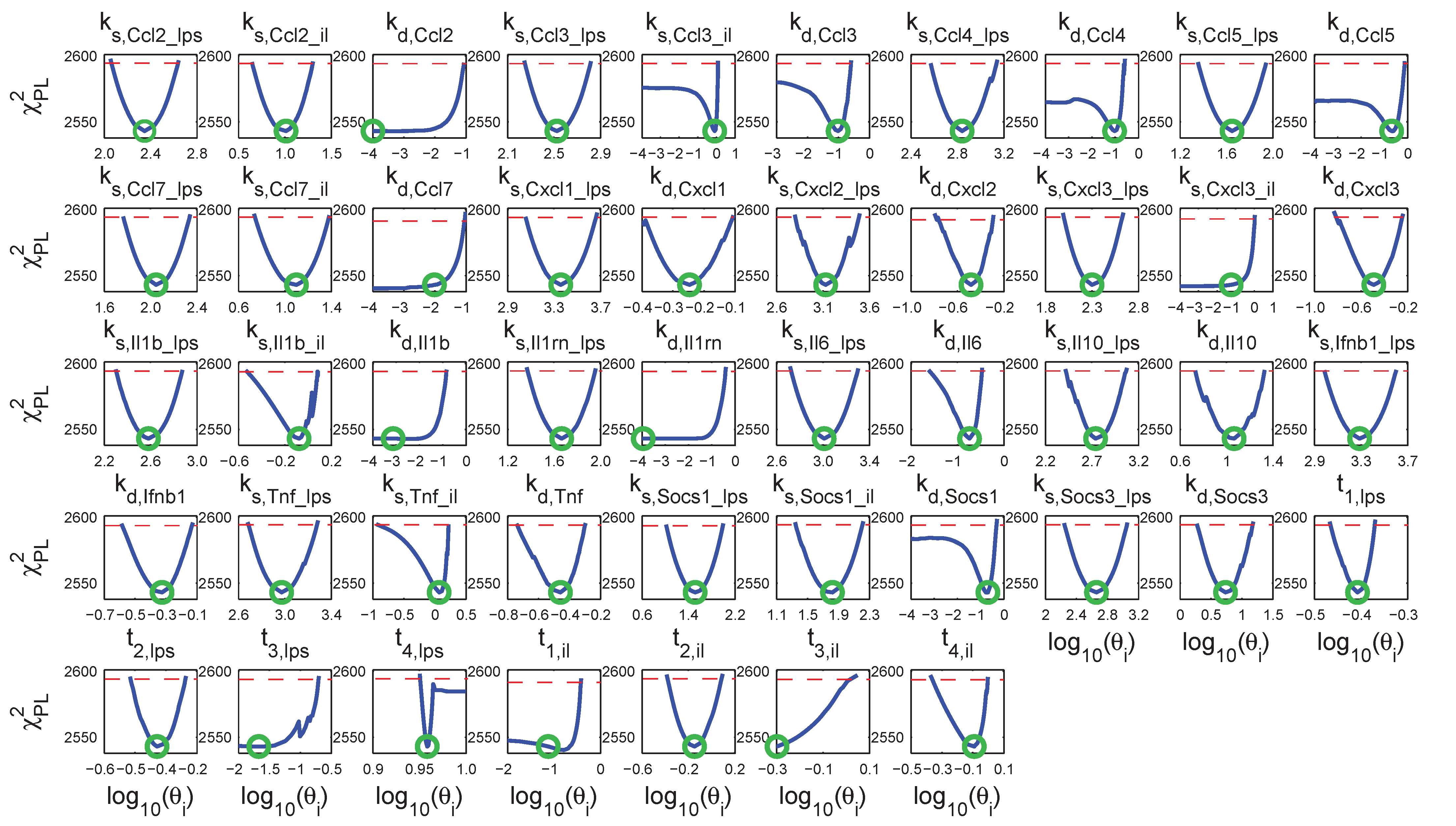

Supplement: S5 Fig — Profile likelihood is displayed by blue lines, estimated parameter values are indicated by green circles. The threshold for 68% confidence intervals is depicted as red line. Parameter values on the base 10 logarithmic x-axis are displayed in orders of magnitude. The χ2PL value is calculated by the sum of mean square errors. For calculation of each profile likelihood, the respective parameter value is decreased and increased based on the estimated optimal value in various steps whereas all other parameter values are again optimized by weighted least-square minimization. If the profile likelihood crosses the threshold line, the parameter is considered as identifiable. Otherwise, the upper or lower bound of the parameter remains unidentifiable. (TIF) [file pcbi.1005018.s005.tif]

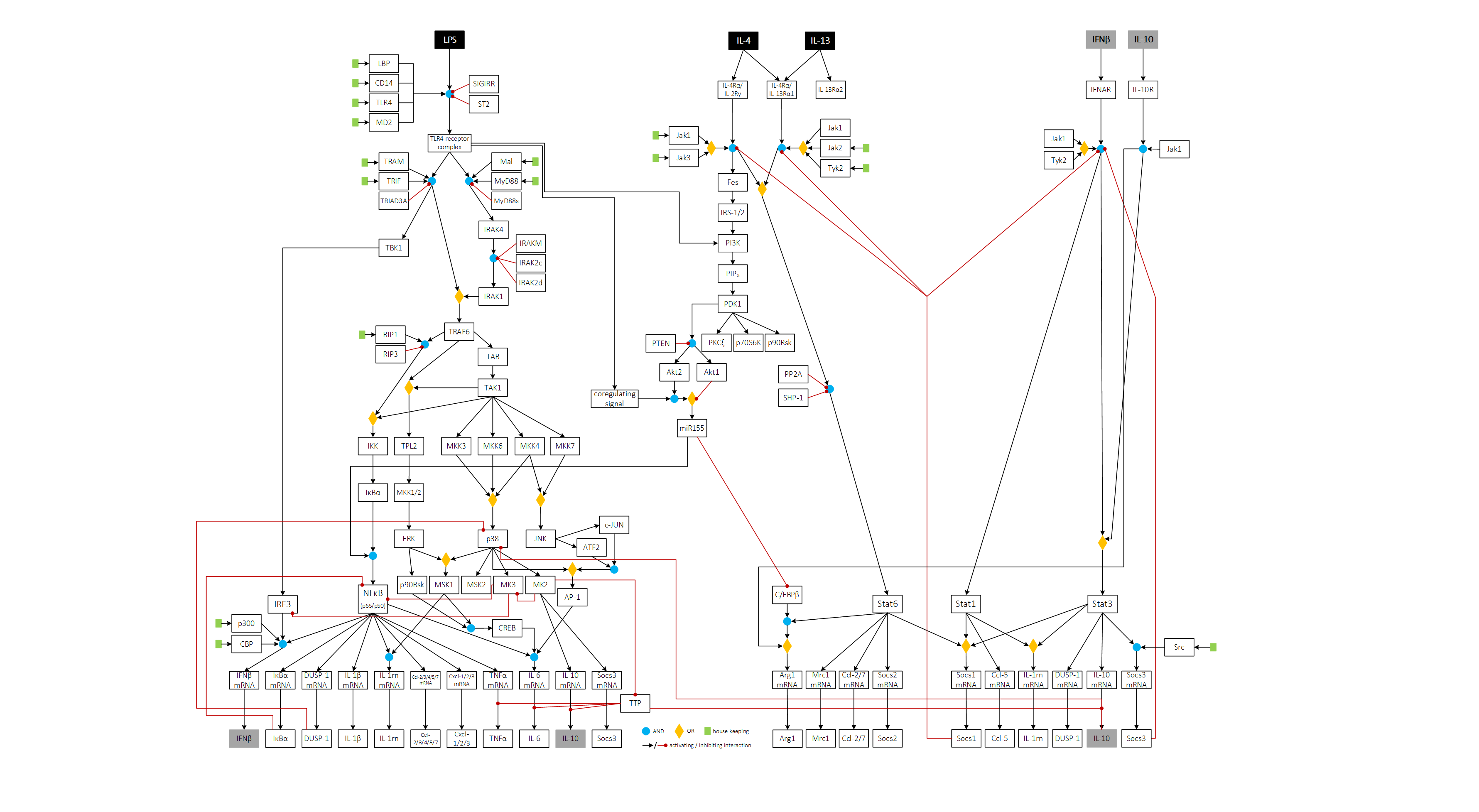

Supplement: S2 Protocol — The file contains the refined Boolean model of macrophage activation implemented with CellNetAnalyzer [50]. CNA is freely available for academic use. Software and online manual can be downloaded from http://www.mpi-magdeburg.mpg.de/projects/cna/cna.html. After starting CNA, a new signal-flow project has to be created using the provided folder ‘MacrophageActivation’. Textboxes are optimized for width 0.007, height 0.015, and font size 8. (ZIP) [file pcbi.1005018.s011.zip › S2_Protocol/MacrophageActivation/ModelScheme.png]
