# Supplementary material for: Model-Based Characterization of Inflammatory Gene Expression Patterns of Activated Macrophages
Source: PLoS Comput Biol. 2016 Jul 27;12(7):e1005018. doi: 10.1371/journal.pcbi.1005018 (PMC4963125; doi:10.1371/journal.pcbi.1005018)
Supplement: S1 Table — Notation of all species of the Boolean model (Fig 11) with their official full name and gene ID according to the NCBI gene data base or description. An asterisk (*) in the last column indicates nodes that are newly added after comparison of experimental data and simulation results of the first version of the Boolean model (S1 Fig). A hash (#) indicates a modified node; the corresponding species in S2 Table is also marked with a hash. (PDF) [file pcbi.1005018.s006.pdf]

No	node	official full name / description	NCBI Gene ID	new
1	Akt1	thymoma viral proto-oncogene 1	11651	
2	Akt2	thymoma viral proto-oncogene 2	11652	
3	AP1	transcription factor consisting of cJun and ATF-2	-	
4	Arg1	arginase, liver	11846	
5	Arg1mRNA	mRNA encoding Arg1	-	
6	ATF2	activating transcription factor 2	11909	
7	CBP	CREB binding protein	12914	
8	Ccl2/3/4/5/6/7	chemokine (C-C motif) ligand 2/3/4/5/7 induced by NF-κB	20296, 20302-4, 20306	#1
9	Ccl2/3/4/5/6/7mRNA	mRNA encoding Ccl2/3/4/5/7 (NF-κB target)	-	#2
10	Ccl2/7mRNA_Stat6	mRNA encoding Ccl2/7 (Stat6 target)	-	#3
11	Ccl2/7_Stat6	chemokine (C-C motif) ligand 2/7 induced by Stat6	20296, 20306	#4
12	Ccl5mRNA_Stat1	mRNA encoding Ccl5 (Stat1 target)	x	
13	Ccl5_Stat1	chemokine (C-C motif) ligand 5 induced by Stat1	20304	
14	CD14	CD14 antigen	12475	
15	CEBPb	CCAAT/enhancer binding protein (C/EBP), beta	12608	
16	cJun	jun proto-oncogene	16476	
17	coSignal	coregulating signal induced by LPS	-	
18	CREB	cAMP responsive element binding protein 1	12912	
19	Cxcl1/2/3	chemokine (C-X-C motif) ligand 1/2/3	14825, 20310, 330122	#5
20	Cxcl1/2/3mRNA	mRNA encoding Cxcl1/2/3	-	#6
21	dum_DUSP1_inh	inhibitory dummy species to decouple activating from inhibitory signals at $\tau = 15$	-	
22	dum_IkBa_inh	inhibitory dummy species to decouple activating from inhibitory signals at $\tau = 15$	-	
23	dum_Socs1_inh	inhibitory dummy species to decouple activating from inhibitory signals at $\tau = 15$	-	
24	dum_Socs3_inh	inhibitory dummy species to decouple activating from inhibitory signals at $\tau = 15$	-	
25	DUSP1mRNA_NFkB	mRNA encoding DUSP1 (NF-κB target)	-	
26	DUSP1mRNA_Stat3	mRNA encoding DUSP1 (Stat3 target)	-	
27	DUSP1_NFkB	dual specificity phosphatase 1 (NF-κB target)	19252	
28	DUSP1_Stat3	dual specificity phosphatase 1 (Stat3 target)	19252	
29	ERK	mitogen-activated protein kinase 1	26413	
30	Fes	feline sarcoma oncogene	14159	
31	hk	housekeeping dummy species activating constantly expressed proteins at $\tau = 0$	-	
32	hk_inh	housekeeping inhibitory dummy species maintaining inhibitory species at $\tau = 15$	-	

No	node	official full name / description	NCBI Gene ID	new
33	hk_medium	housekeeping dummy species maintaining secreted proteins	-	
34	IFNAR	interferon (alpha and beta) receptor 1	15975	
35	IFNbmRNA	mRNA encoding IFNβ	-	
36	IFNb_medium	interferon beta 1 (secreted)	15977	
37	IFNb_syn	interferon beta 1 (newly synthesized)	15977	
38	IkBa	nuclear factor of kappa light polypeptide gene enhancer in B cells inhibitor, alpha	18035	
39	IkBamRNA	mRNA encoding IκBα	-	
40	IkBa_syn	IκBα (newly synthesized)	15977	
41	IKK	inhibitor of kappa B kinase	-	
42	IL10mRNA_Stat3	mRNA encoding IL-10 (Stat3 target)	-	#7
43	IL10mRNA_MK2	mRNA encoding IL-10 (induced by MK2)	-	*
44	IL10R	interleukin 10 receptor consisting of alpha and beta subunit	16154/5	
45	IL10_medium	interleukin 10 (secreted)	16153	
46	IL10_syn_MK2	interleukin 10 (newly synthesized, induced by MK2)	16153	*
47	IL10_syn_Stat3	interleukin 10 (newly synthesized, Stat3 target)	16153	#8
48	IL13	interleukin 13	16163	
49	IL13Ra2	interleukin 13 receptor, alpha 2	16165	
50	IL1bmRNA	mRNA encoding IL-1β	-	
51	IL1b_syn	interleukin 1 beta (newly synthesized)	16176	
52	IL1rnmRNA_NFkB	mRNA encoding IL1rn (NF-κB target)	-	#9
53	IL1rnmRNA_Stat	mRNA encoding IL1rn (Stat target)	-	*
54	IL1rn_NFkB	interleukin 1 receptor antagonist (NF-κB target)	16181	#10
55	IL1rn_Stat	interleukin 1 receptor antagonist (Stat target)	16181	*
56	IL4	interleukin 4	16189	
57	IL4Ra/IL13Ra1	receptor complex consisting of interleukin 4 receptor, alpha and interleukin 13 receptor, alpha 1	16190, 16164	
58	IL4Ra/IL2Ry	receptor complex consisting of interleukin 4 receptor, alpha and interleukin 2 receptor, gamma chain	16190, 16186	
59	IL6mRNA	mRNA encoding IL-6	-	
60	IL6_syn	interleukin 6 (newly synthesized)	16193	
61	IRAK1	interleukin-1 receptor-associated kinase 1	16179	
62	IRAK2c	interleukin-1 receptor-associated kinase 2	108960	
63	IRAK2d	interleukin-1 receptor-associated kinase 2	108960	
64	IRAK4	interleukin-1 receptor-associated kinase 4	266632	
65	IRAKM	interleukin-1 receptor-associated kinase 3	73914	
66	IRF3	interferon regulatory factor 3	54131	
67	IRS1/2	insulin receptor substrate 1/2	16367, 384783	
68	Jak1	Janus kinase 1	16451	
69	Jak2	Janus kinase 2	16452	

No	node	official full name / description	NCBI Gene ID	new
70	Jak3	Janus kinase 3	16453	
71	JNK	mitogen-activated protein kinase 8/9	26419/20	
72	LBP	lipopolysaccharide binding protein	16803	
73	LPS	lipopolysaccharide	-	
74	Mal	myelin and lymphocyte protein, T cell differentiation protein	17153	
75	MD2	lymphocyte antigen 96	17087	
76	miR155	microRNA 155	387173	
77	MK2	MAP kinase-activated protein kinase 2	17164	
78	MK3	MAP kinase-activated protein kinase 3	102626	
79	MKK1/2	mitogen-activated protein kinase kinase 1/2	26395/6	
80	MKK3	mitogen-activated protein kinase kinase 3	26397	
81	MKK4	mitogen-activated protein kinase kinase 4	26398	
82	MKK6	mitogen-activated protein kinase kinase 6	26399	
83	MKK7	mitogen-activated protein kinase kinase 7	26400	
84	Mrc1	mannose receptor, C type 1	17533	
85	Mrc1mRNA	mRNA encoding Mrc1	-	
86	MSK1	mitogen- and stress-activated kinase 1	-	
87	MSK2	mitogen- and stress-activated kinase 2	-	
88	MyD88	myeloid differentiation primary response gene 88	17874	
89	MyD88s	myeloid differentiation primary response gene 88 short (alternative splice variant)	17874	
90	NFkB	nuclear factor of kappa light polypeptide gene enhancer in B cells	-	
91	p300	E1A binding protein p300	328572	
92	p38	mitogen-activated protein kinase 14	26416	
93	p70S6K	ribosomal protein S6 kinase	72508	
94	p90Rsk_il	ribosomal protein S6 kinase	20111	
95	p90Rsk_lps	ribosomal protein S6 kinase	20111	
96	PDK1	pyruvate dehydrogenase kinase, isoenzyme 1	228026	
97	PI3K	phosphatidylinositol 3-kinase	-	
98	PIP3	phosphatidylinositol 3 phosphat	-	
99	PKC	protein kinase C	-	
100	PP2A	protein phosphatase 2	-	
101	PTEN	phosphatase and tensin homolog	19211	
102	RIP1	receptor (TNFRSF)-interacting serine-threonine kinase 1	19766	
103	RIP3	receptor (TNFRSF)-interacting serine-threonine kinase 3	56532	
104	SHP1	protein tyrosine phosphatase, non-receptor type 6	15170	
105	SIGIRR	single immunoglobulin and toll-interleukin 1 receptor (TIR) domain	24058	
106	Socs1	suppressor of cytokine signaling 1	12703	
107	Socs1mRNA	mRNA encoding Socs1	-	

No	node	official full name / description	NCBI Gene ID	new
108	Socs2	suppressor of cytokine signaling 2	216233	
109	Socs2mRNA	mRNA encoding Socs2	-	
110	Socs3mRNA_MK2	mRNA encoding Socs3 (induced by MK2)	-	*
111	Socs3mRNA_Stat3	mRNA encoding Socs3 (Stat3 target)	-	#11
112	Socs3_MK2	suppressor of cytokine signaling 3 (induced by MK2)	12702	*
113	Socs3_Stat3	suppressor of cytokine signaling 3 (Stat3 target)	12702	#12
114	Src	Rous sarcoma oncogene	20779	
115	ST2	interleukin 1 receptor-like 1	17082	
116	Stat1	signal transducer and activator of transcription 1	20846	
117	Stat3	signal transducer and activator of transcription 3	20848	
118	Stat6	signal transducer and activator of transcription 6	20852	
119	TAB	TGFβ activated kinase 1/MAP3K7 binding protein	68652, 66724	
120	TAK1	TGFβ activated kinase 1/MAP3K7	26409	
121	TBK1	TANK-binding kinase 1	56480	
122	TLR4	toll-like receptor 4	21898	
123	TLR4RC	TLR4 receptor complex consiting of TLR4, CD14, LPS, LBP, MD2	-	
124	TNFamRNA	mRNA encoding TNFα	-	
125	TNFa_syn	tumor necrosis factor	21926	
126	TPL2	mitogen-activated protein kinase kinase kinase 8	26410	
127	TRAF6	TNF receptor-associated factor 6	22034	
128	TRAM	toll-like receptor adaptor molecule 2	225471	
129	TRIAD3A	an E3 ubiquitin-protein ligase regulating Toll-like receptors	-	
130	TRIF	toll-like receptor adaptor molecule 1	106759	
131	TTP	tristetraprolin/zinc finger protein 36	22695	
132	Tyk2	tyrosine kinase 2	54721	
