# Supplementary material for: Model-Based Characterization of Inflammatory Gene Expression Patterns of Activated Macrophages
Source: PLoS Comput Biol. 2016 Jul 27;12(7):e1005018. doi: 10.1371/journal.pcbi.1005018 (PMC4963125; doi:10.1371/journal.pcbi.1005018)
Supplement: S2 Table — List of all species that were part of the first literature-based version of the Boolean model (S1 Fig) but were modified or deleted after comparison with experimental data and thus do not occur in the final model version (S1 Table). A hash (#) indicates a modified node; the corresponding species in S1 Table is also marked with a hash. (PDF) [file pcbi.1005018.s007.pdf]

No	node	official full name / description	NCBI Gene ID	mod
1	Ccl2/3/4/5	chemokine (C-C motif) ligand 2/3/4/5 induced by NF-κB	20296, 20302-4	#1
2	Ccl2/3/4/5mRNA	mRNA encoding Ccl2/3/4/5 (NF-κB target)	-	#2
3	Ccl2mRNA_Stat6	mRNA encoding Ccl2 (Stat6 target)	-	#3
4	Ccl2_Stat6	chemokine (C-C motif) ligand 2 induced by Stat6	20296	#4
5	Cxcl1/2/3/5	chemokine (C-X-C motif) ligand 1/2/3/5	14825, 20310, 330122, 20311	#5
6	Cxcl1/2/3/5mRNA	mRNA encoding Cxcl1/2/3/5	-	#6
7	gp130	interleukin 6 signal transducer	16195	
8	gp80	interleukin 6 receptor, alpha	16194	
9	IFNamRNA	mRNA encoding IFNα	-	
10	IFNa_medium	interferon alpha 1 (secreted)	15962	
11	IFNa_syn	interferon alpha 1 (newly synthesized)	15962	
12	IFNgmRNA	mRNA encoding IFNγ	-	
13	IFNGR	interferon gamma receptor 1	15979	
14	IFNg_medium	interferon gamma (secreted)	15978	
15	IFNg_syn	interferon gamma (newly synthesized)	15978	
16	IL10mRNA	mRNA encoding IL-10	-	#7
17	IL10_syn	interleukin 10 (newly synthesized)	16153	#8
18	IL1bRC	IL-1β receptor complex consisting of IL-1β, IL1R1 and IL1RAcP	-	
19	IL1b_medium	interleukin 1 beta (secreted)	16176	
20	IL1R1	interleukin 1 receptor, type I	16177	
21	IL1RAcP	interleukin 1 receptor accessory protein	16180	
22	IL1rnmRNA	mRNA encoding IL1rn	-	#9
23	IL1rn	interleukin 1 receptor antagonist	16181	#10
24	IL6RC	IL-6 receptor complex consisting of IL-6, gp80 and gp130	-	
25	IL6_medium	interleukin 6 (secreted)	16193	
26	OSM	oncostatin M	18413	
27	OSMmRNA	mRNA encoding OSM	-	
28	Socs3mRNA	mRNA encoding Socs3	-	#11
29	Socs3	suppressor of cytokine signaling 3	12702	#12
30	TNFaRC1	TNF receptor complex 1 consisting of TNF, TNFR1, TRADD, Rip1 and TRAF2	-	
31	TNFa_medium	tumor necrosis factor (secreted)	21926	
32	TNFR1	tumor necrosis factor receptor superfamily, member 1	21937/8	
33	TRADD	TNFRSF1A-associated via death domain	71609	
34	TRAF2	TNF receptor-associated factor 2	22030	
