# Supplementary material for: Model-Based Characterization of Inflammatory Gene Expression Patterns of Activated Macrophages
Source: PLoS Comput Biol. 2016 Jul 27;12(7):e1005018. doi: 10.1371/journal.pcbi.1005018 (PMC4963125; doi:10.1371/journal.pcbi.1005018)
Supplement: S3 Table — List of all logical equations of the Boolean model (Fig 11) in the notation of the CellNetAnalyzer with their assigned timescale constant τ, a short description and reference. An asterisk (*) in the last column indicates equations that are newly added after comparison of experimental data and simulation results of the first version of the Boolean model (S1 Fig). A hash (#) indicates a modified equation; the corresponding interaction equation in S4 Table is also marked with a hash. (PDF) [file pcbi.1005018.s008.pdf]

No	equation	τ	description	reference	new
1	hk = CBP	0	basal expression	-	
2	hk = CD14	0	basal expression	-	
3	hk = Jak1	0	basal expression	-	
4	hk = Jak2	0	basal expression	-	
5	hk = Jak3	0	basal expression	-	
6	hk = LBP	0	basal expression	-	
7	hk = Mal	0	basal expression	-	
8	hk = MD2	0	basal expression	-	
9	hk = MyD88	0	basal expression	-	
10	hk = p300	0	basal expression	-	
11	hk = RIP1	0	basal expression	-	
12	hk = Src	0	basal expression	-	
13	hk = TLR4	0	basal expression	-	
14	hk = TRAM	0	basal expression	-	
15	hk = TRIF	0	basal expression	-	
16	hk = Tyk2	0	basal expression	-	
17	!hk = PP2A	0	basal expression	-	
18	!hk = PTEN	0	basal expression	-	
19	!hk = RIP3	0	basal expression	-	
20	!hk = SHP1	0	basal expression	-	
21	LBP + CD14 + TLR4 + MD2 + !SIGIRR + !ST2 + LPS = TLR4RC	1	early TLR4 signaling	[1, 2]	
22	TLR4RC + Mal + MyD88 + !MyD88s = IRAK4	1	early TLR4 signaling	[1, 2]	
23	TLR4RC = coSignal	1	early TLR4 signaling	-	
24	TLR4RC = PI3K	1	early TLR4 signaling	[3, 4]	
25	IRAK4 + !IRAKM + !IRAK2c + !IRAK2d = IRAK1	1	early TLR4 signaling	[1, 2]	
26	IRAK1 = TRAF6	1	early TLR4 signaling	[1, 2]	
27	TRAF6 = TAB	1	early TLR4 signaling	[1, 2]	
28	TRAF6 = TPL2	1	early TLR4 signaling	[1, 5]	
29	TAB = TAK1	1	early TLR4 signaling	[1, 2]	
30	TAK1 = IKK	1	early TLR4 signaling	[1, 2]	
31	TAK1 = TPL2	1	early TLR4 signaling	[1, 5]	
32	TAK1 = MKK3	1	early TLR4 signaling	[6, 7, 2]	
33	TAK1 = MKK4	1	early TLR4 signaling	[7]	
34	TAK1 = MKK6	1	early TLR4 signaling	[6, 7]	
35	TAK1 = MKK7	1	early TLR4 signaling	[7, 2]	
36	TPL2 = MKK1/2	1	early TLR4 signaling	[5]	
37	MKK1/2 = ERK	1	early TLR4 signaling	[1, 5]	
38	MKK3 + !dum_DUSP1_inh = p38	1	early TLR4 signaling	[6, 7, 2]	
39	MKK4 + !dum_DUSP1_inh = p38	1	early TLR4 signaling	[6, 7]	
40	MKK6 + !dum_DUSP1_inh = p38	1	early TLR4 signaling	[6, 7]	
41	MKK4 = JNK	1	early TLR4 signaling	[6, 8]	
42	MKK7 = JNK	1	early TLR4 signaling	[6, 8, 2]	
43	ERK = p90Rsk_lps	1	early TLR4 signaling	[5]	
44	ERK = MSK1	1	early TLR4 signaling	[5]	
45	p38 = MSK1	1	early TLR4 signaling	[6, 9]	
46	p38 = MSK2	1	early TLR4 signaling	[6]	

No	equation	τ	description	reference	new
47	p38 = MK2	1	early TLR4 signaling	[6, 9, 10]	
48	p38 = AP1	1	early TLR4 signaling	[11]	
49	JNK = cJun	1	early TLR4 signaling	[8]	
50	JNK = ATF2	1	early TLR4 signaling	[8]	
51	cJun + ATF2 = AP1	1	early TLR4 signaling	[8]	
52	MSK1 + p90Rsk_lps = CREB	1	early TLR4 signaling	[5, 9]	
53	!MK2 = MK3	1	early TLR4 signaling	[6]	
54	!MK2 = TTP	1	early TLR4 signaling	[6, 9, 10]	
55	IKK = IkBa	1	early TLR4 signaling	[1, 2]	
56	IkBa + !dum_IkBa_inh + !MK3 + miR155 = NFkB	1	early TLR4 signaling	[1, 12, 13, 14, 2, 15]	
57	IL4 = IL4Ra/IL2Ry	1	IL-4/13 signaling	[16]	
58	IL4 = IL4Ra/IL13Ra1	1	IL-4/13 signaling	[16]	
59	IL13 = IL4Ra/IL13Ra1	1	IL-4/13 signaling	[16]	
60	IL13 = IL13Ra2	1	IL-4/13 signaling	[16]	
61	IL4Ra/IL2Ry + Jak1 + !PP2A + !SHP1 + !dum_Socs1_inh = Stat6	1	IL-4/13 signaling	[17, 18, 19]	
62	IL4Ra/IL2Ry + Jak3 + !PP2A + !SHP1 + !dum_Socs1_inh = Stat6	1	IL-4/13 signaling	[17, 18, 19]	
63	IL4Ra/IL13Ra1 + Jak1 + !PP2A + !SHP1 + !dum_Socs1_inh = Stat6	1	IL-4/13 signaling	[17, 18, 19]	
64	IL4Ra/IL13Ra1 + Jak2 + !PP2A + !SHP1 + !dum_Socs1_inh = Stat6	1	IL-4/13 signaling	[17, 18, 19]	
65	IL4Ra/IL13Ra1 + Tyk2 + !PP2A + !SHP1 + !dum_Socs1_inh = Stat6	1	IL-4/13 signaling	[17, 18, 19]	
66	IL4Ra/IL2Ry + Jak1 = Fes	1	IL-4/13 signaling	[19, 20]	
67	IL4Ra/IL2Ry + Jak3 = Fes	1	IL-4/13 signaling	[19, 20]	
68	Fes = IRS1/2	1	PI3K/Akt signaling	[20, 21]	
69	IRS1/2 = PI3K	1	PI3K/Akt signaling	[20, 21]	
70	PI3K = PIP3	1	PI3K/Akt signaling	[21, 22]	
71	PIP3 = PDK1	1	PI3K/Akt signaling	[21, 22]	
72	PDK1 = PKC	1	PI3K/Akt signaling	[23, 22]	
73	PDK1 = p70S6K	1	PI3K/Akt signaling	[23, 21]	
74	PDK1 = p90Rsk_il	1	PI3K/Akt signaling	[20]	
75	PDK1 + !PTEN = Akt1	1	PI3K/Akt signaling	[24, 21, 25, 22]	
76	PDK1 + !PTEN = Akt2	1	PI3K/Akt signaling	[24, 21, 25, 22]	
77	!Akt1 = miR155	1	PI3K/Akt signaling	[26, 15]	
78	Akt2 + coSignal = miR155	1	PI3K/Akt signaling	[26, 15]	
79	!miR155 = CEBPb	1	PI3K/Akt signaling	[26, 15]	
80	IFNb_medium = IFNAR	1	type I IFN signaling	[6, 27]	
81	IFNAR + Jak1 + !dum_Socs1_inh = Stat1	1	type I IFN signaling	[19, 27]	
82	IFNAR + Tyk2 + !dum_Socs1_inh = Stat1	1	type I IFN signaling	[19, 27]	
83	IFNAR + Jak1 + !dum_Socs3_inh = Stat3	1	type I IFN signaling	[19, 27]	
84	IFNAR + Tyk2 + !dum_Socs3_inh = Stat3	1	type I IFN signaling	[19, 27]	
85	IL10_medium = IL10R	1	IL-10 signaling	[6, 28]	
86	Jak1 + IL10R = Stat3	1	IL-10 signaling	[6, 28]	
87	TLR4RC + TRAM + TRIF + !TRIAD3A = TRAF6	2	late TLR4 signaling	[1, 29, 2]	

No	equation	τ	description	reference	new
88	TLR4RC + TRAM + TRIF + !TRIAD3A = TBK1	2	late TLR4 signaling	[1, 2]	
89	TRAF6 + RIP1 + !IL1rn = IKK	2	late TLR4 signaling	[1, 2]	
90	TBK1 + !MK3 = IRF3	2	late TLR4 signaling	[1, 30, 29, 2]	
91	NFkB + IRF3 + p300 + CBP = IFNbmRNA	5	TLR4 signaling	[6, 31]	
92	NFkB = IkBamRNA	5	TLR4 signaling	[1, 2]	
93	NFkB = DUSP1mRNA_NFkB	5	TLR4 signaling	[32]	
94	NFkB = IL1bmRNA	5	TLR4 signaling	[1, 6]	
95	NFkB + MSK1 = IL1rnmRNA_NFkB	5	TLR4 signaling	[6, 33]	#1
96	NFkB = Ccl2/3/4/5/7mRNA	5	TLR4 signaling	[14]	#2
97	NFkB = Cxcl1/2/3mRNA	5	TLR4 signaling	[14]	#3
98	NFkB + AP1 + CREB = IL6mRNA	5	TLR4 signaling	[1, 8]	
99	NFkB = TNFamRNA	5	TLR4 signaling	[1]	
100	MK2 = IL10mRNA_MK2	5	TLR4 signaling	data	*
101	MK2 = Socs3mRNA_MK2	5	TLR4 signaling	data	*
102	Stat6 + CEBPb= Arg1mRNA	5	IL-4/13 signaling	[14, 15]	
103	Stat6 = Mrc1mRNA	5	IL-4/13 signaling	[14]	
104	Stat6 = Ccl2/7mRNA_Stat6	5	IL-4/13 signaling	data, [14]	#4
105	Stat6 = Socs1mRNA	5	IL-4/13 signaling	[17]	
106	Stat6 = Socs2mRNA	5	IL-4/13 signaling	[17, 34]	
107	Stat1 = IL1rnmRNA_Stat	5	IFNβ signaling	data	*
108	Stat1 = Socs1mRNA	5	IFNβ signaling	[35]	
109	Stat1 = Ccl5mRNA_Stat1	5	IFNβ signaling	[14]	
110	Stat3 = IL10mRNA_Stat3	5	IFN/IL-10 signaling	[6]	
111	Stat3 + Src = Socs3mRNA_Stat3	5	IFN/IL-10 signaling	[6, 36, 37]	#5
112	Stat3 = Socs1mRNA	5	IFN/IL-10 signaling	[35]	
113	Stat3 = DUSP1mRNA_Stat3	5	IFN/IL-10 signaling	[6, 37]	
114	Stat3 = IL1rnmRNA_Stat	5	IFN/IL-10 signaling	data	*
115	IL10R = Arg1mRNA	5	IL-10 signaling	[38]	
116	IFNbmRNA = IFNb_syn	7	TLR4 signaling	[6, 31]	
117	IkBabmRNA = IkBa_syn	7	TLR4 signaling	[1, 2]	
118	DUSP1mRNA_NFkB = DUSP1_NFkB	7	TLR4 signaling	[6, 32]	
119	IL1bmRNA = IL1b_syn	7	TLR4 signaling	[1, 6]	
120	IL1rnmRNA_NFkB = IL1rn_NFkB	7	TLR4 signaling	[6, 33]	#6
121	Ccl2/3/4/5/7mRNA = Ccl2/3/4/5/7	7	TLR4 signaling	[14]	#7
122	Cxcl1/2/3mRNA = Cxcl1/2/3	7	TLR4 signaling	[14]	#8
123	IL6mRNA + !TTP = IL6_syn	7	TLR4 signaling	[1, 6]	
124	TNFamRNA + !TTP = TNFa	7	TLR4 signaling	[1, 6, 9, 10]	
125	IL10mRNA_MK2 + !TTP = IL10_MK2	7	TLR4 signaling	[39, 6]	*
126	Socs3mRNA_MK2 = Socs3_MK2	7	TLR4 signaling	-	*
127	Arg1mRNA = Arg1	7	IL-4/13 signaling	[14]	
128	Mrc1mRNA = Mrc1	7	IL-4/13 signaling	[14]	
129	Ccl2/7mRNA_Stat6 = Ccl2/7_Stat6	7	IL-4/13 signaling	[14]	#9
130	Socs2mRNA = Socs2	7	IL-4/13 signaling	[34]	
131	Socs1mRNA = Socs1	7	IL/IFNβ signaling	[34]	
132	Ccl5mRNA_Stat1 = Ccl5_Stat1	7	IFN/IL-10 signaling	[14]	
133	IL1rnmRNA_Stat = IL1rn_Stat	7	IFN/IL-10 signaling	[14]	*

No	equation	τ	description	reference	new
134	DUSP1mRNA_Stat3 = DUSP1_Stat3	7	IFN/IL-10 signaling	[32, 6]	
135	IL10mRNA_Stat3 + !TTP = IL10_syn_Stat	7	IFN/IL-10 signaling	[6]	#10
136	Socs3mRNA_Stat3 = Socs3_Stat3	7	IFN/IL-10 signaling	[34]	#11
137	IFNb_syn = IFNb_medium	10	protein secretion	[6]	
138	IL10_syn_MK2 = IL10_medium	10	protein secretion	[6]	*
139	IL10_syn_Stat3 = IL10_medium	12	protein secretion	[6]	#12
140	hk_medium + LPS = IFNb_medium	15	maintain secreted proteins	-	
141	hk_medium + LPS = IL10_medium	15	maintain secreted proteins	-	
142	DUSP1_NFkB = dum_DUSP1_inh	15	activation of the inhibitory dummy species	-	
143	DUSP1_Stat3 = dum_DUSP1_inh	15	activation of the inhibitory dummy species	-	
144	hk_inh + LPS = dum_DUSP1_inh	15	maintain inhibitory species at $\tau = 15$	-	
145	IkBa = dum_IkBa_inh	15	activation of the inhibitory dummy species	-	
146	hk_inh + LPS = dum_IkBa_inh	15	maintain inhibitory species at $\tau = 15$	-	
147	Socs1 = dum_Socs1_inh	15	activation of the inhibitory dummy species	-	
148	hk_inh + LPS = dum_Socs1_inh	15	maintain inhibitory species at $\tau = 15$	-	
149	hk_inh + IL4 = dum_Socs1_inh	15	maintain inhibitory species at $\tau = 15$	-	
150	Socs3_MK2 = dum_Socs3_inh	15	activation of the inhibitory dummy species	-	*
151	Socs3_Stat3 = dum_Socs3_inh	15	activation of the inhibitory dummy species	-	#13
152	hk_inh + LPS = dum_Socs3_inh	15	maintain inhibitory species at $\tau = 15$	-	

References

[1] Akira S, Takeda K. Toll-like receptor signalling. Nature reviews Immunology. 2004 jul;4(7):499–511.

[2] Pålsson-McDermott EM, O'Neill LaJ. Signal transduction by the lipopolysaccharide receptor, Toll-like receptor-4. Immunology. 2004 oct;113(2):153–162.

[3] McGuire VA, Gray A, Monk CE, Santos SG, Lee K, Aubareda A, et al. Cross talk between the Akt and p38α pathways in macrophages downstream of Toll-like receptor signaling. Molecular and cellular biology. 2013 nov;33(21):4152–65.

[4] Monick MM, Carter aB, Robeff PK, Flaherty DM, Peterson MW, Hunninghake GW. Lipopolysaccharide activates Akt in human alveolar macrophages resulting in nuclear accumulation and transcriptional activity of beta-catenin. Journal of immunology (Baltimore, Md : 1950). 2001 apr;166(7):4713–20.

[5] Eliopoulos AG, Dumitru CD, Wang CC, Cho J, Tsichlis PN. Induction of COX-2 by LPS in macrophages is regulated by Tpl2-dependent CREB activation signals. EMBO Journal. 2002;21(18):4831–4840.

[6] Bode JG, Ehlting C, Häussinger D. The macrophage response towards LPS and its control through the p38 MAPK-STAT3 axis. Cellular Signalling. 2012;24(6):1185–1194.

[7] Cargnello M, Roux PP. Activation and function of the MAPKs and their substrates, the MAPK-activated protein kinases. Microbiology and molecular biology reviews : MMBR. 2011;75(1):50–83.

[8] Hambleton J, Weinstein SL, Lem L, DeFranco AL. Activation of c-Jun N-terminal kinase in bacterial lipopolysaccharide-stimulated macrophages. Proceedings of the National Academy of Sciences of the United States of America. 1996;93(7):2774–2778.

[9] Ronkina N, Menon MB, Schwermann J, Arthur JSC, Legault H, Telliez JB, et al. Stress induced gene expression: a direct role for MAPKAP kinases in transcriptional activation of immediate early genes. Nucleic acids research. 2011 apr;39(7):2503–18.

[10] Tiedje C, Ronkina N, Tehrani M, Dhamija S, Laass K, Holtmann H, et al. The p38/MK2-driven exchange between tristetraprolin and HuR regulates AU-rich element-dependent translation. PLoS genetics. 2012 sep;8(9):e1002977.

[11] Ono K, Han J. The p38 signal transduction pathway Activation and function. Cellular Signalling. 2000;12:1–13.

[12] Guha M, Mackman N. The phosphatidylinositol 3-kinase-Akt pathway limits lipopolysaccharide activation of signaling pathways and expression of inflammatory mediators in human monocytic cells. Journal of Biological Chemistry. 2002;277:32124–32132.

[13] Hoeflich KP, Luo J, Rubie Ea, Tsao MS, Jin O, Woodgett JR. Requirement for glycogen synthase kinase-3beta in cell survival and NF-kappaB activation. Nature. 2000;406(July):86–90.

[14] Mantovani A, Sica A, Sozzani S, Allavena P, Vecchi A, Locati M. The chemokine system in diverse forms of macrophage activation and polarization. Trends in Immunology. 2004;25(12):677–686.

[15] Zhou D, Huang C, Lin Z, Zhan S, Kong L, Fang C, et al. Macrophage polarization and function with emphasis on the evolving roles of coordinated regulation of cellular signaling pathways. Cellular signalling. 2014 feb;26(2):192–7.

[16] Kelly-Welch AE, Hanson EM, Boothby MR, Keegan AD. Interleukin-4 and interleukin-13 signaling connections maps. Science (New York, NY). 2003;300(June):1527–1528.

[17] Dickensheets H, Vazquez N, Sheikh F, Gingras S, Murray PJ, Ryan JJ, et al. Suppressor of cytokine signaling-1 is an IL-4-inducible gene in macrophages and feedback inhibits IL-4 signaling. Genes and immunity. 2007;8:21–27.

[18] Hebenstreit D, Wirnsberger G, Horejs-Hoeck J, Duschl A. Signaling mechanisms, interaction partners, and target genes of STAT6. Cytokine and Growth Factor Reviews. 2006;17:173–188.

[19] Heller NM, Qi X, Junttila IS, Shirey KA, Vogel SN, Paul WE, et al. Type I IL-4Rs selectively activate IRS-2 to induce target gene expression in macrophages. Science signaling. 2008;1(December 2008):ra17.

[20] Jiang H, Harris MB, Rothman P. IL-4/IL-13 signaling beyond JAK/STAT. Journal of Allergy and Clinical Immunology. 2000;105:1063–1070.

[21] Kelly-Welch A, Hanson EM, Keegan AD. Interleukin-4 (IL-4) pathway. Science's STKE : signal transduction knowledge environment. jul;(293):cm9.

[22] Okkenhaug K. Signaling by the phosphoinositide 3-kinase family in immune cells. Annual review of immunology. 2013;31:675–704.

[23] Cantley LC. The phosphoinositide 3-kinase pathway. Science (New York, NY). 2002;296(2002):1655–1657.

[24] Cantley LC, Neel BG. New insights into tumor suppression: PTEN suppresses tumor formation by restraining the phosphoinositide 3-kinase/AKT pathway. Proceedings of the National Academy of Sciences of the United States of America. 1999;96(April):4240–4245.

[25] Lawlor Ma, Alessi DR. PKB/Akt: a key mediator of cell proliferation, survival and insulin responses? Journal of cell science. 2001;114:2903–2910.

[26] Arranz A, Doxaki C, Vergadi E, Martinez de la Torre Y, Vaporidi K, Lagoudaki ED, et al. Akt1 and Akt2 protein kinases differentially contribute to macrophage polarization. Proceedings of the National Academy of Sciences of the United States of America. 2012 jun;109(24):9517–22.

[27] Platanias LC. Mechanisms of type-I- and type-II-interferon-mediated signalling. Nature reviews Immunology. 2005 may;5(5):375–386.

[28] Riley JK, Takeda K, Akira S, Schreiber RD. Interleukin-10 receptor signaling through the JAK-STAT pathway. Requirement for two distinct receptor-derived signals for anti-inflammatory action. Journal of Biological Chemistry. 1999;274(23):16513–16521.

[29] Fitzgerald Ka, Rowe DC, Barnes BJ, Caffrey DR, Visintin A, Latz E, et al. LPS-TLR4 signaling to IRF-3/7 and NF-kappaB involves the toll adapters TRAM and TRIF. The Journal of experimental medicine. 2003;198(7):1043–1055.

[30] Ehlting C, Ronkina N, Böhmer O, Albrecht U, Bode KA, Lang KS, et al. Distinct functions of the Mitogen-activated Protein Kinase-activated Protein (MAPKAP) kinases MK2 and MK3: MK2 mediates lipopolysaccharide-induced Signal Transducers and Activators of Transcription 3 (STAT3) activation by preventing negative regulatory ef. Journal of Biological Chemistry. 2011;286(27):24113–24124.

[31] Smith PL, Lombardi G, Foster GR. Type I interferons and the innate immune response - More than just antiviral cytokines. Molecular Immunology. 2005;42:869–877.

[32] Hammer M, Mages J, Dietrich H, Schmitz F, Striebel F, Murray PJ, et al. Control of dual-specificity phosphatase-1 expression in activated macrophages by IL-10. European Journal of Immunology. 2005;35:2991–3001.

[33] Darragh J, Ananieva O, Courtney A, Elcombe S, Arthur JSC. MSK1 regulates the transcription of IL-1ra in response to TLR activation in macrophages. The Biochemical journal. 2010;425:595–602.

[34] Wilson HM. SOCS proteins in macrophage polarization and function. Frontiers in Immunology. 2014;5(July):1–5.

[35] Niemand C, Nimmesgern A, Haan S, Fischer P, Schaper F, Rossaint R, et al. Activation of STAT3 by IL-6 and IL-10 in primary human macrophages is differentially modulated by suppressor of cytokine signaling 3. Journal of immunology (Baltimore, Md : 1950). 2003 mar;170(6):3263–3272.

[36] Murray PJ, Allen JE, Biswas SK, Fisher Ea, Gilroy DW, Goerdt S, et al. Macrophage Activation and Polarization: Nomenclature and Experimental Guidelines. Immunity. 2014;41(1):14–20.

[37] Sabat R, Grütz G, Warszawska K, Kirsch S, Witte E, Wolk K, et al. Biology of interleukin-10. Cytokine and Growth Factor Reviews. 2010;21:331–344.

[38] Schreiber T, Ehlers S, Heitmann L, Rausch A, Mages J, Murray PJ, et al. Autocrine IL-10 induces hallmarks of alternative activation in macrophages and suppresses antituberculosis effector mechanisms without compromising T cell immunity. Journal of immunology (Baltimore, Md : 1950). 2009 jul;183(2):1301–12.

[39] Saraiva M, Christensen JR, Tsytsykova AV, Goldfeld AE, Ley SC, Kioussis D, et al. Identification of a macrophage-specific chromatin signature in the IL-10 locus. Journal of immunology (Baltimore, Md : 1950). 2005;175:1041–1046.
