# Supplementary material for: Model-Based Characterization of Inflammatory Gene Expression Patterns of Activated Macrophages
Source: PLoS Comput Biol. 2016 Jul 27;12(7):e1005018. doi: 10.1371/journal.pcbi.1005018 (PMC4963125; doi:10.1371/journal.pcbi.1005018)
Supplement: S4 Table — List of all logical equations that were part of the first literature-based version of the Boolean model (S1 Fig) but were modified or deleted after comparison with experimental data and thus do not occur in the final model version (S3 Table). A hash (#) indicates a modified equation; the corresponding interaction in S3 Table is also marked with a hash. (PDF) [file pcbi.1005018.s009.pdf]

No	equation	τ	description	reference	mod
1	hk = IL1R1	0	IL-1β signaling	-	
2	hk = IL1RAcP	0	IL-1β signaling	-	
3	hk = TNFR1	0	TNFα signaling	-	
4	hk = TRADD	0	TNFα signaling	-	
5	hk = TRAF2	0	TNFα signaling	-	
6	hk = gp80	0	IL-6 signaling	-	
7	hk = gp130	0	IL-6 signaling	-	
8	IL1b_medium + IL1R1 + IL1RAcP = IL1bRC	1	IL-1β signaling	[1]	
9	MyD88 + !MyD88s + IL1bRC = IRAK4	1	IL-1β signaling	[1]	
10	TNFa_medium + RIP1 + TNFR1 + TRADD + TRAF2 = TNFaRC1	1	TNFα signaling	[1]	
11	TNFaRC1 = TAK1	1	TNFα signaling	[1]	
12	IL6_medium + gp80 + gp130 = IL6RC	1	IL-6 signaling	[2]	
13	IL6RC + Jak1 + !dum_Socs3_inh = Stat3	1	IL-6 signaling	[2, 3]	
14	IFNg_medium = IFNGR	1	IFNγ signaling	[2, 3]	
15	IFNGR + Jak1 + !dum_Socs1_inh = Stat1	1	IFNγ signaling	[4]	
16	IFNGR + Jak2 + !dum_Socs1_inh = Stat1	1	IFNγ signaling	[4]	
17	IFNa_medium = IFNAR	1	IFNα signaling	[4]	
18	NFkB + IRF3 + p300 + CBP = IFNgmRNA	5	TLR4 signaling	[5]	
19	NFkB + IRF3 + p300 + CBP = IFNamRNA	5	TLR4 signaling	[6]	
20	NFkB + MSK1 = IL1rnmRNA	5	TLR4 signaling	[7, 8]	#1
21	NFkB = OSMmRNA	5	TLR4 signaling	[7, 9]	
22	NFkB = Ccl2/3/4/5mRNA	5	TLR4 signaling	[10]	#2
23	NFkB = Cxcl1/2/3/5mRNA	5	TLR4 signaling	[10]	#3
24	Stat6 = Ccl2mRNA	5	IL-4/13 signaling	[10]	#4
25	Stat3 + Src = Socs3mRNA	5	IFN/IL-10 signaling	[7, 11, 12]	#5
26	IFNgmRNA = IFNg_syn	7	TLR4 signaling	[5]	
27	IFNamRNA = IFNa_syn	7	TLR4 signaling	[6]	
28	IL1rnmRNA = IL1rn	7	TLR4 signaling	[7, 8]	#6
29	OSMmRNA = OSM	7	TLR4 signaling	[7, 9]	
30	Ccl2/3/4/5mRNA = Ccl2/3/4/5	7	TLR4 signaling	[10]	#7
31	Cxcl1/2/3/5mRNA = Cxcl1/2/3/5	7	TLR4 signaling	[10]	#8
32	Ccl2mRNA_Stat6 = Ccl2_Stat6	7	IL-4/13 signaling	[10]	#9
33	IL10mRNA + !TTP = IL10_syn	7	IFN/IL-10 signaling	[7]	#10
34	Socs3mRNA = Socs3	7	IFN/IL-10 signaling	[13]	#11
35	IL1b_syn = IL1b_medium	10	protein secretion	[7, 14]	
36	TNFa_syn = TNFa_medium	10	protein secretion	[7, 14]	
37	IL6_syn = IL6_medium	10	protein secretion	[7]	
38	IFNg_syn = IFNg_medium	10	protein secretion	[5]	
39	IFNa_syn = IFNa_medium	10	protein secretion	[6]	
40	IL10_syn = IL10_medium	12	protein secretion	[7]	#12
41	hk_medium + LPS = IL1b_medium	15	maintain secreted proteins	-	
42	hk_medium + LPS = TNFa_medium	15	maintain secreted proteins	-	
43	hk_medium + LPS = IL6_medium	15	maintain secreted proteins	-	
44	hk_medium + LPS = IFNg_medium	15	maintain secreted proteins	-	

No	equation	τ	description	reference	mod
45	hk_medium + LPS = IFNa_medium	15	maintain secreted proteins	-	
46	Socs3 = dum_Socs3_inh	15	activation of the inhibitory dummy species	-	#13

References

[1] Verstrepen L, Bekaert T, Chau TL, Tavernier J, Chariot A, Beyaert R. TLR-4, IL-1R and TNF-R signaling to NF-kappaB: variations on a common theme. Cellular and molecular life sciences : CMLS. 2008 oct;65(19):2964–78.

[2] Aaronson DS, Horvath CM. A road map for those who don't know JAK-STAT. Science (New York, NY). 2002 may;296(5573):1653–1655.

[3] Heinrich PC, Behrmann I, Haan S, Hermanns HM, Müller-Newen G, Schaper F. Principles of interleukin (IL)-6-type cytokine signalling and its regulation. The Biochemical journal. 2003 aug;374(Pt 1):1–20.

[4] Platanias LC. Mechanisms of type-I- and type-II-interferon-mediated signalling. Nature reviews Immunology. 2005 may;5(5):375–386.

[5] Fultz MJ, Barber Sa, Dieffenbach CW, Vogel SN. Induction of IFN-gamma in macrophages by lipopolysaccharide. International immunology. 1993 nov;5(11):1383–1392.

[6] Fitzgerald Ka, Rowe DC, Barnes BJ, Caffrey DR, Visintin A, Latz E, et al. LPS-TLR4 signaling to IRF-3/7 and NF-kappaB involves the toll adapters TRAM and TRIF. The Journal of experimental medicine. 2003;198(7):1043–1055.

[7] Bode JG, Ehlting C, Häussinger D. The macrophage response towards LPS and its control through the p38 MAPK-STAT3 axis. Cellular Signalling. 2012;24(6):1185–1194.

[8] Darragh J, Ananieva O, Courtney A, Elcombe S, Arthur JSC. MSK1 regulates the transcription of IL-1ra in response to TLR activation in macrophages. The Biochemical journal. 2010;425:595–602.

[9] Wallace PM, MacMaster JF, Rouleau Ka, Brown TJ, Loy JK, Donaldson KL, et al. Regulation of inflammatory responses by oncostatin M. Journal of immunology (Baltimore, Md : 1950). 1999;162:5547–5555.

[10] Mantovani A, Sica A, Sozzani S, Allavena P, Vecchi A, Locati M. The chemokine system in diverse forms of macrophage activation and polarization. Trends in Immunology. 2004;25(12):677–686.

[11] Murray PJ, Allen JE, Biswas SK, Fisher Ea, Gilroy DW, Goerdt S, et al. Macrophage Activation and Polarization: Nomenclature and Experimental Guidelines. Immunity. 2014;41(1):14–20.

[12] Sabat R, Grütz G, Warszawska K, Kirsch S, Witte E, Wolk K, et al. Biology of interleukin-10. Cytokine and Growth Factor Reviews. 2010;21:331–344.

[13] Wilson HM. SOCS proteins in macrophage polarization and function. Frontiers in Immunology. 2014;5(July):1–5.

[14] Ulich TR, Watson LR, Yin SM, Guo KZ, Wang P, Thang H, et al. The intratracheal administration of endotoxin and cytokines. I. Characterization of LPS-induced IL-1 and TNF mRNA expression and the LPS-, IL-1-, and TNF-induced inflammatory infiltrate. The American journal of pathology. 1991;138(6):1485–1496.
