# Supplementary material for: Model-Based Characterization of Inflammatory Gene Expression Patterns of Activated Macrophages
Source: PLoS Comput Biol. 2016 Jul 27;12(7):e1005018. doi: 10.1371/journal.pcbi.1005018 (PMC4963125; doi:10.1371/journal.pcbi.1005018)
Supplement: S1 Protocol — (PDF) [file pcbi.1005018.s010.pdf]

Supporting Information

Model-based characterization of inflammatory gene expression patterns of activated macrophages

Julia Sanwald[1*], Ute Albrecht[2*], Christian Ehlting[2*], Maria Thomas[3], Ulrich M. Zanger[3], Oliver Sawodny[1], Dieter Häussinger[2], Michael Ederer[1], Ronny Feuer[1#], Johannes G. Bode[2#]

[1] Institute for System Dynamics, University of Stuttgart, 70511 Stuttgart, Germany

[2] Clinic of Gastroenterology, Hepatology and Infectious Diseases, Heinrich-Heine-University, 40225 Düsseldorf, Germany

[3] Dr. Margarete Fischer-Bosch Institute of Clinical Pharmacology, 70376 Stuttgart, and University of Tübingen, 72076 Tübingen, Germany

[*],[#] contributed equally

Contents

1 Data Analysis **1**

1.1 Outlier Detection 2

1.2 Kernel Density Estimation 2

2 Dynamic Modeling **5**

2.1 Model Setup 5

2.2 Model Equations 7

2.3 Parameter Values 10

1 Data Analysis

The Fluidigm data set was analyzed using LEMming, a multivariable statistics approach [1]. Briefly, a linear error model was used to estimate (1) the probe pipetting error per assay (PPEpA), (2) the treatment effect per mouse (TEpM), (3) the sample pipetting error (SPE) and (4) the treatment effect per gene (TEpG).

A single measurement Y is given by

$$Y = PPEpA + TEpM + SPE + TEpG + \epsilon \tag{1}$$

with the remaining error ϵ comprising non-systematic technical errors and biological variance. The estimated technical errores $\tilde{Y} = PPEpA + SPE$ are discarded and the estimated variable is given by

$$\hat{Y} = TEpM + TEpG + \epsilon \tag{2}$$

Expression values of genes that are regulated by the respective treatment are expressed as fold of untreated control cells

$$\hat{Y}' = -\Big(\hat{Y} - Mean(\hat{Y}_{untreated})\Big) \tag{3}$$

The Fluidigm data set comprises four assays à 48 genes as well as 48, 38, 43, and 22 samples. Deducting the genes interleukin 17A (Il17a), interleukin 28B (Il28b), and interferon γ (Ifng) that were close to our beyond the detection limit in most of the measurements, this results in a total number of 6075 measurement points of 45 genes including the two control genes beta-actin (Actb) and glyceraldehyde-3-phosphate dehydrogenase (Gapdh).

1.1 Outlier Detection

Measurement points that lay outside the box plot with 1.5 IQR (Interquartile range) whiskers are classified as outliers (Fig. S5) and were discarded.

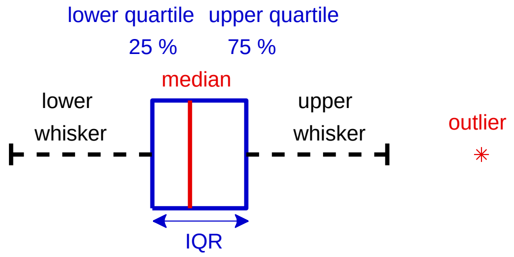

Figure S5: **Box-whisker plot**. The box, indicated by a blue rectangle, contains 50 % of the data, the median is indicated by a red line within the box. The lower and upper border of the box represents the lower (first, 25 %) and upper (third, 75 %) quartile, respectively. The length of the box is termed interquartile range (IQR). The whiskers display the data points outside the box but do not exceed 1.5 times the IQR. All data points that lay outside the whiskers are classified as outliers.

$$Out_{lower} < q_{25} - 1.5 \cdot IQR \tag{4}$$

$$Out_{upper} > q_{75} + 1.5 \cdot IQR \tag{5}$$

From the complete Fluidigm data set, 249 outliers were deleted resulting in 5826 measurement points.

1.2 Kernel Density Estimation

Kernel density estimation is conducted based on the Fluidigm data set to select genes that are clearly up- or downregulated. Gaussian kernel is assumed and the probability density function of each stimulation, gene, and time point is calculated if at least three independent measurements per time point exist. Then, for each time point (0.5, 1, 2, 6, 10 h) the area of intersection with the probability density function of untreated cells (0 h) is calculated and genes are ranked in ascending order by the smallest area of intersection

for each stimulation. For genes that are classified as clearly regulated and non-regulated a representative example is shown in Fig. S6.

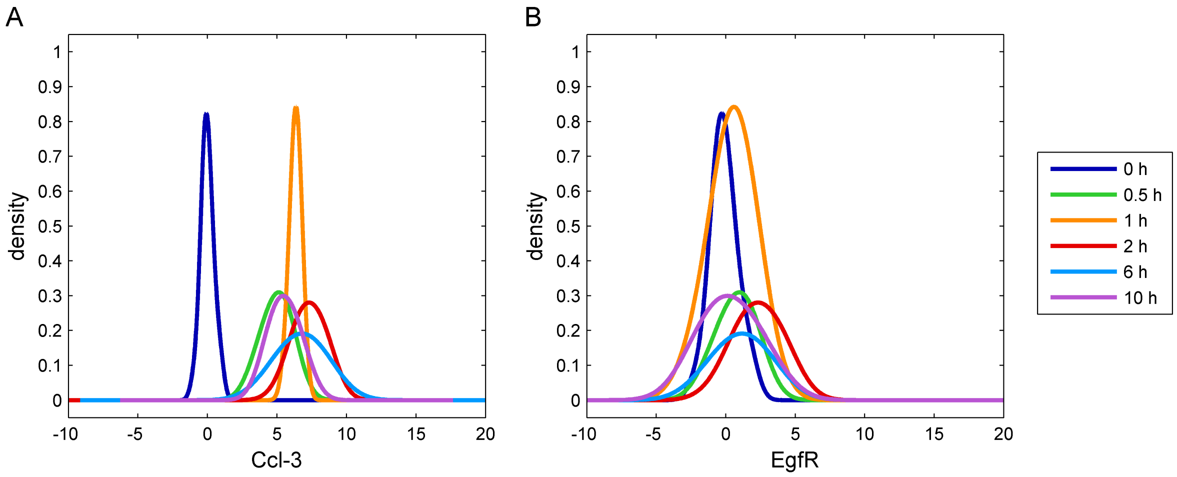

Figure S6: **Kernel density estimates for two representative genes**. Examples of kernel density estimates of genes that were classified as (A) clearly regulated upon LPS stimulation such as Ccl-3 and (B) non-regulated like EgfR.

Since Gapdh is measured as control gene that is supposed to be constantly expressed during the treatment, all genes that have a larger area of intersection than Gapdh are discarded. The ranking of the other genes is shown in Fig. S7. All genes whose minimal intersection area is smaller than those of Gapdh and which are at east 4-fold and 1.5-fold regulated upon LPS and IL-4/13 stimulation, respectively, are selected for model integration and marked red in Fig. S7. Genes marked blue are signature genes for M1 and M2 macrophages that are not inlcuded in the ODE model but are checked to ensure accurate activation of macrophages. Upon LPS stimulation, the genes Cd14, Cd69 and Cd86 are upregulated as expected (Fig. S7 A). The M2 marker genes Arg1 and Mrc1 are not regulated and downregulated, respectively. After stimulation with IL-4 and IL-13, Arg1 and Mrc1 are upregulated as awaited (Fig. S7 B). The M1 signature genes Cd14 and Cd69 are downregulated and Cd86 is almost not regulated with a maximal mean of 0.93 over all measuerd time points confirming correct activation of macrophages.

By this approach, 16 and 7 genes were selected for model integration for M1 and M2 macrophages, respectively.

A

Gene	Min Area	Time Point	Mean
Cxcl2	-7.03E-12	1 h	8.34
Tnf	-3.71E-12	1 h	7.58
Ccl4	3.06E-15	2 h	8.75
Ccl3	2.20E-13	1 h	7.31
Il1rn	1.08E-10	10 h	8.87
Il1b	5.30E-10	1 h	7.58
Tnfrsf1b	1.52E-08	10 h	3.53
Cxcl1	8.98E-07	1 h	9.58
Ccl5	2.22E-06	2 h	7.61
Il10	3.96E-06	1 h	6.04
Cd69	7.32E-05	1 h	7.30
Cd14	1.17E-04	1 h	3.51
Cxcl3	1.15E-03	2 h	6.98
Ccl2	3.35E-03	2 h	7.53
Il6	4.00E-03	1 h	9.38
Ccl7	4.21E-03	6 h	6.37
Met	7.41E-03	10 h	3.22
Mrc1	1.41E-02	10 h	4.97
Cd86	1.54E-02	6 h	2.70
Socs3	4.35E-02	10 h	6.92
Osm	5.95E-02	0.5 h	2.37
Ifnb1	6.01E-02	2 h	9.68
Tnfrsf1a	6.06E-02	10 h	1.32
Ccr3	6.10E-02	1 h	3.16
Ifnar2	6.30E-02	10 h	1.19
Socs1	9.26E-02	10 h	6.51
Ifnar1	9.36E-02	0.5 h	1.19
Ccr5	1.15E-01	10 h	1.42
Gapdh	1.16E-01	10 h	2.12

B

Gene	Min Area	Time Point	Mean
Mrc1	5.44E-02	2 h	2.10
Cd14	1.34E-01	6 h	-1.66
Ccr5	1.88E-01	2 h	-1.24
Ccl7	2.00E-01	1 h	3.58
Tnf	2.15E-01	1 h	-1.65
Socs1	2.45E-01	10 h	5.19
Il6st	2.60E-01	6 h	1.15
Cd69	2.66E-01	10 h	-2.43
Tgfb1	2.75E-01	6 h	0.63
Ccl2	2.77E-01	1 h	3.29
Cd86	2.99E-01	6 h	0.93
Ccl3	3.10E-01	2 h	-1.55
Ccr2	3.22E-01	10 h	-1.13
Il10	3.91E-01	6 h	1.20
Il1b	3.95E-01	6 h	-1.98
Arg1	3.97E-01	1 h	6.29
Socs3	4.00E-01	0.5	1.23
Cxcl3	4.39E-01	10 h	-1.87
Ifnar2	4.40E-01	10 h	0.25
Ccl4	4.46E-01	0.5	1.03
Hgf	4.61E-01	6 h	-1.35
Cxcl2	4.62E-01	6 h	-0.73
Osm	4.73E-01	6 h	0.28
Il10ra	4.83E-01	1 h	-0.68
Egf	4.89E-01	6 h	-0.67
Il10rb	4.96E-01	6 h	0.28
Tnfrsf1a	5.04E-01	1 h	0.07
Gapdh	5.40E-01	6 h	0.41

Figure S7: **Ranking of genes for model integration**. Genes are ranked in ascending order by the smalles area of intersection of the pdf (probability density function) of treated vs. pdf of untreated cells for (A) LPS stimulation and (B) IL-4/13 stimulation. Genes marked red are selected for model integration. Genes marked blue are marker genes, i.e. Cd14, Cd69, Cd86 for M1 macrophages and Arg1, Mrc1 for M2 macrophages. For each gene, the minimal area of intersection with untreated controls is displayed in the second column. The corresponding time point is shown in the third column. The maximal or minimal mean for up- and downregulated genes, respectively, is displayed in the forth column.

2 Dynamic Modeling

2.1 Model Setup

The model is based on ordinary differential equations (ODEs) and implemented using the MATLAB® Toolbox PottersWheel [2]. Model setup is explained in [3]. Briefly, to reduce complexity of the system, the signaling path upstream of transcription factor (TF) activation is neglected and grouped together into an approximated rectangular function $u(t)$ representing the input of the model (illustrated in Fig. S8)

$$u(t) = \frac{1}{2}\Big(tanh\big(s\cdot(t-t_1)\big) - tanh\big(s\cdot(t-t_2)\big)\Big), \quad t_1 > 0, t_2 > t_1 \tag{6}$$

with slope coefficient s that is set to 1000 to approximate a rectangular function and time constants t_1 and t_2 determining the beginning and end of transcription factor activity and are inferred from experimental data.

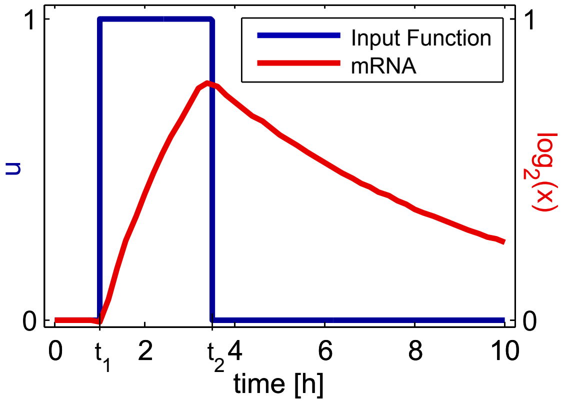

Figure S8: **Implementation of mRNA expression in dependence of the input function**. The input function u (blue) represents transcription factor activity and is implemented by an approximated rectangular function having values between 0 and 1 for non-activated and activated TF, respectively. The TF regulates mRNA expression (red), i.e. induced mRNA synthesis is active only while the TF is activated (u = 1).

The relative mRNA expression levels x_{mRNA} are given by

$$\dot{x}_{\mathrm{mRNA}} = k_b + k_s * u(t) - k_d * x_{\mathrm{mRNA}}, \quad x_{\mathrm{mRNA}}(0) = 1 \tag{7}$$

with rate constant for basal synthesis k_b, induced synthesis following transcription factor activation k_s and degradation k_d. Initial values for mRNA levels equal one due to normalization of experimental data on untreated control cells.

At $t = 0$ the system is at steady state and it is

$$\frac{k_{s,basal}}{k_d} = x_{\mathrm{mRNA}}(0) = 1 \tag{8}$$

reducing the number of parameters by one per gene.

2.2 Model Equations

M1 macrophages

- Genes regulated by NF-κB

$$\dot{x}_{\text{Ccl2}} = k_{d,\text{Ccl2}} - k_{d,\text{Ccl2}} * x_{\text{Ccl2}} + k_{s,\text{Ccl2\_lps}} * u_1(t) \tag{9}$$

$$\dot{x}_{\text{Ccl3}} = k_{d,\text{Ccl3}} - k_{d,\text{Ccl3}} * x_{\text{Ccl3}} + k_{s,\text{Ccl3\_lps}} * u_1(t) \tag{10}$$

$$\dot{x}_{\text{Ccl4}} = k_{d,\text{Ccl4}} - k_{d,\text{Ccl4}} * x_{\text{Ccl4}} + k_{s,\text{Ccl4\_lps}} * u_1(t) \tag{11}$$

$$\dot{x}_{\text{Ccl7}} = k_{d,\text{Ccl7}} - k_{d,\text{Ccl7}} * x_{\text{Ccl7}} + k_{s,\text{Ccl7\_lps}} * u_1(t) \tag{12}$$

$$\dot{x}_{\text{Cxcl1}} = k_{d,\text{Cxcl1}} - k_{d,\text{Cxcl1}} * x_{\text{Cxcl1}} + k_{s,\text{Cxcl1\_lps}} * u_1(t) \tag{13}$$

$$\dot{x}_{\text{Cxcl2}} = k_{d,\text{Cxcl2}} - k_{d,\text{Cxcl2}} * x_{\text{Cxcl2}} + k_{s,\text{Cxcl2\_lps}} * u_1(t) \tag{14}$$

$$\dot{x}_{\text{Cxcl3}} = k_{d,\text{Cxcl3}} - k_{d,\text{Cxcl3}} * x_{\text{Cxcl3}} + k_{s,\text{Cxcl3\_lps}} * u_1(t) \tag{15}$$

$$\dot{x}_{\text{Il1b}} = k_{d,\text{Il1b}} - k_{d,\text{Il1b}} * x_{\text{Il1b}} + k_{s,\text{Il1b\_lps}} * u_1(t) \tag{16}$$

$$\dot{x}_{\text{Il6}} = k_{d,\text{Il6}} - k_{d,\text{Il6}} * x_{\text{Il6}} + k_{s,\text{Il6\_lps}} * u_1(t) \tag{17}$$

$$\dot{x}_{\text{Ifnb1}} = k_{d,\text{Ifnb1}} - k_{d,\text{Ifnb1}} * x_{\text{Ifnb1}} + k_{s,\text{Ifnb1\_lps}} * u_1(t) \tag{18}$$

$$\dot{x}_{\text{Tnf}} = k_{d,\text{Tnf}} - k_{d,\text{Tnf}} * x_{\text{Tnf}} + k_{s,\text{Tnf\_lps}} * u_1(t) \tag{19}$$

- Genes regulated by NF-κB and Stat3

$$\dot{x}_{\text{Ccl5}} = k_{d,\text{Ccl5}} - k_{d,\text{Ccl5}} * x_{\text{Ccl5}} + k_{s,\text{Ccl5\_lps}} * u_2(t) \tag{20}$$

$$\dot{x}_{\text{Il1rn}} = k_{d,\text{Il1rn}} - k_{d,\text{Il1rn}} * x_{\text{Il1rn}} + k_{s,\text{Il1rn\_lps}} * u_2(t) \tag{21}$$

$$\dot{x}_{\text{Il10}} = k_{d,\text{Il10}} - k_{d,\text{Il10}} * x_{\text{Il10}} + k_{s,\text{Il10\_lps}} * u_2(t) \tag{22}$$

$$\dot{x}_{\text{Socs3}} = k_{d,\text{Socs3}} - k_{d,\text{Socs3}} * x_{\text{Socs3}} + k_{s,\text{Socs3\_lps}} * u_2(t) \tag{23}$$

- Genes regulated by Stat3

$$\dot{x}_{\text{Socs1}} = k_{d,\text{Socs1}} - k_{d,\text{Socs1}} * x_{\text{Socs1}} + k_{s,\text{Socs1\_lps}} * u_3(t) \tag{24}$$

M2 macrophages

- Genes upregulated in response to IL-4/13 stimulation by Stat6

$$\dot{x}_{\text{Ccl2}} = k_{d,\text{Ccl2}} - k_{d,\text{Ccl2}} * x_{\text{Ccl2}} + k_{s,\text{Ccl2\_il}} * u_4(t) \tag{25}$$

$$\dot{x}_{\text{Ccl7}} = k_{d,\text{Ccl7}} - k_{d,\text{Ccl7}} * x_{\text{Ccl7}} + k_{s,\text{Ccl7\_il}} * u_4(t) \tag{26}$$

$$\dot{x}_{\text{Socs1}} = k_{d,\text{Socs1}} - k_{d,\text{Socs1}} * x_{\text{Socs1}} + k_{s,\text{Socs1\_il}} * u_4(t) \tag{27}$$

- Genes downregulated in response to IL-4/13 stimulation

$$\dot{x}_{\text{Il1b}} = k_{d,\text{Il1b}} - k_{d,\text{Il1b}} * x_{\text{Il1b}} + k_{s,\text{Il1b\_il}} * u_5(t) \tag{28}$$

$$\dot{x}_{\text{Tnf}} = k_{d,\text{Tnf}} - k_{d,\text{Tnf}} * x_{\text{Tnf}} + k_{s,\text{Tnf\_il}} * u_5(t) \tag{29}$$

$$\dot{x}_{\text{Ccl3}} = k_{d,\text{Ccl3}} - k_{d,\text{Ccl3}} * x_{\text{Ccl3}} + k_{s,\text{Ccl3\_il}} * u_5(t) \tag{30}$$

$$\dot{x}_{\text{Cxcl3}} = k_{d,\text{Cxcl3}} - k_{d,\text{Cxcl3}} * x_{\text{Cxcl3}} + k_{s,\text{Cxcl3\_il}} * u_5(t) \tag{31}$$

Input Functions

$$u_1(t) = \frac{1}{2}\Bigg(tanh\Big(s\cdot(t-t_{1,\mathrm{lps}})\Big) - tanh\Big(s\cdot(t-t_{2,\mathrm{lps}})\Big)\Bigg) \tag{32}$$

$$\begin{aligned} u_2(t) = \ & \frac{1}{2}\Bigg(tanh\Big(s\cdot(t-t_{1,\mathrm{lps}})\Big) - tanh\Big(s\cdot(t-t_{2,\mathrm{lps}})\Big) \\ & + tanh\Big(s\cdot(t-t_{3,\mathrm{lps}})\Big) - tanh\Big(s\cdot(t-t_{4,\mathrm{lps}})\Big)\Bigg) \end{aligned} \tag{33}$$

$$u_3(t) = \frac{1}{2}\Bigg(tanh\Big(s\cdot(t-t_{3,\mathrm{lps}})\Big) - tanh\Big(s\cdot(t-t_{4,\mathrm{lps}})\Big)\Bigg) \tag{34}$$

$$u_4(t) = \frac{1}{2}\Bigg(tanh\Big(s\cdot(t-t_{1,\mathrm{il}})\Big) - tanh\Big(s\cdot(t-t_{2,\mathrm{il}})\Big)\Bigg) \tag{35}$$

$$u_5(t) = -\frac{1}{2}\Bigg(tanh\Big(s\cdot(t-t_{3,\mathrm{il}})\Big) - tanh\Big(s\cdot(t-t_{4,\mathrm{il}})\Big)\Bigg) \tag{36}$$

Time Constants for Beginning and End of Transcription Factor Activity

$$t_{1,\mathrm{lps}} = a_{\mathrm{lps}} \tag{37}$$

$$t_{2,\mathrm{lps}} = a_{\mathrm{lps}} + b_{\mathrm{lps}} \tag{38}$$

$$t_{3,\mathrm{lps}} = a_{\mathrm{lps}} + b_{\mathrm{lps}} + c_{\mathrm{lps}} \tag{39}$$

$$t_{4,\mathrm{lps}} = a_{\mathrm{lps}} + b_{\mathrm{lps}} + c_{\mathrm{lps}} + d_{\mathrm{lps}} \tag{40}$$

$$t_{1,\mathrm{il}} = a_{\mathrm{il}} \tag{41}$$

$$t_{2,\mathrm{il}} = a_{\mathrm{il}} + b_{\mathrm{il}} \tag{42}$$

$$t_{3,\mathrm{il}} = c_{\mathrm{il}} \tag{43}$$

$$t_{4,\mathrm{il}} = c_{\mathrm{il}} + d_{\mathrm{il}} \tag{44}$$

2.3 Parameter Values

- Synthesis rate constants

Parameter	Value [h^{-1}]	Lower CI (PL)	Upper CI (PL)
$k_{s,\text{Ccl2\_lps}}$	223.03	115.90	432.44
$k_{s,\text{Ccl3\_lps}}$	336.22	177.61	654.52
$k_{s,\text{Ccl4\_lps}}$	694.68	371.09	1375.1
$k_{s,\text{Ccl5\_lps}}$	44.48	22.76	87.10
$k_{s,\text{Ccl7\_lps}}$	111.72	58.46	216.64
$k_{s,\text{Cxcl1\_lps}}$	2280.56	1134.8	4597.6
$k_{s,\text{Cxcl2\_lps}}$	1350.01	632.23	3077.1
$k_{s,\text{Cxcl3\_lps}}$	200.66	99.47	424.63
$k_{s,\text{Il1b\_lps}}$	383.02	201.18	749.11
$k_{s,\text{Il1rn\_lps}}$	46.53	23.53	90.37
$k_{s,\text{Il6\_lps}}$	1023.04	532.13	1986.5
$k_{s,\text{Il10\_lps}}$	550.39	263.13	1170.1
$k_{s,\text{Ifnb1\_lps}}$	1944.70	969.31	3990.8
$k_{s,\text{Tnf\_lps}}$	937.75	486.80	1893.9
$k_{s,\text{Socs1\_lps}}$	32.86	10.33	98.79
$k_{s,\text{Socs3\_lps}}$	457.17	176.37	1126.6
$k_{s,\text{Ccl2\_il}}$	10.27	4.45	20.00
$k_{s,\text{Ccl3\_il}}$	0.81	0	1.23
$k_{s,\text{Ccl7\_il}}$	12.61	5.53	23.97
$k_{s,\text{Cxcl3\_il}}$	0.06	0	1.06
$k_{s,\text{Il1b\_il}}$	0.84	0.29	1.22
$k_{s,\text{Tnf\_il}}$	1.17	0.12	1.66
$k_{s,\text{Socs1\_il}}$	66.26	22.25	162.11

- Degradation rate constants

Parameter	Value [h^{-1}]	Lower CI (PL)	Upper CI (PL)
$k_{d,\text{Ccl2}}$	1.00E-04	0	0.09
$k_{d,\text{Ccl3}}$	9.77E-02	0	0.25
$k_{d,\text{Ccl4}}$	9.46E-02	0	0.26
$k_{d,\text{Ccl5}}$	0.21	0	0.75
$k_{d,\text{Ccl7}}$	9.68E-03	0	0.09
$k_{d,\text{Cxcl1}}$	0.57	0.39	0.78
$k_{d,\text{Cxcl2}}$	0.33	0.16	0.51
$k_{d,\text{Cxcl3}}$	0.32	0.15	0.57
$k_{d,\text{Il1b}}$	7.41E-04	0	0.15
$k_{d,\text{Il1rn}}$	1.15E-04	0	0.39
$k_{d,\text{Il6}}$	0.18	0.02	0.34
$k_{d,\text{Il10}}$	11.67	5.4	21.26
$k_{d,\text{Ifnb1}}$	0.47	0.26	0.74
$k_{d,\text{Tnf}}$	0.35	0.18	0.50
$k_{d,\text{Socs1}}$	0.21	0	0.49
$k_{d,\text{Socs3}}$	5.42	1.89	14.99

- Time points for beginning and end of transcription factor activity

Parameter	Value [h^{-1}]	Lower CI (PL)	Upper CI (PL)
a_{lps}	0.3915	0.3421	0.4261
b_{lps}	0.3721	0.3059	0.4605
c_{lps}	0.0212	0	0.1968
d_{lps}	9.0883	8.9319	Inf
a_{il}	0.0760	0	0.3836
b_{il}	0.7131	0.4173	1.2178
c_{il}	0.5041	0	1.0391
d_{il}	0.8108	0.4325	0.9934

References

[1] Feuer R, Vlaic S, Arlt J, Sawodny O, Dahmen U, Zanger UM, et al. LEMming: A Linear Error Model to Normalize Parallel Quantitative Real-Time PCR (qPCR) Data as an Alternative to Reference Gene Based Methods. PloS One. 2015;10(9):e0135852.

[2] Maiwald T, Timmer J. Dynamical modeling and multi-experiment fitting with PottersWheel. Bioinformatics. 2008 Sep;24(18):2037–2043.

[3] Sanwald J, Albrecht U, Wagenpfeil J, Thomas M, Sawodny O, Bode JG, et al. Modeling the LPS-induced effects on transcription factor activation and gene expression in murine macrophages. Engineering in Medicine and Biology Society (EMBC), 2015 37th Annual International Conference of the IEEE. 2015 Aug;2015:3989–92.
